# Environmental Antimicrobial Resistance: Implications for Food Safety and Public Health

**DOI:** 10.3390/antibiotics13111087

**Published:** 2024-11-14

**Authors:** Onyinye Victoria Ifedinezi, Nnabueze Darlington Nnaji, Christian Kosisochukwu Anumudu, Chiemerie Theresa Ekwueme, Chijioke Christopher Uhegwu, Francis Chukwuebuka Ihenetu, Promiselynda Obioha, Blessing Oteta Simon, Precious Somtochukwu Ezechukwu, Helen Onyeaka

**Affiliations:** 1 Department of Pharmaceutical Chemistry, University of South Wales, Newport NP20 2BP, UK; 2 School of Chemical Engineering, University of Birmingham, Birmingham B15 2TT, UK; 3 Department of Microbiology, University of Nigeria, Nsukka 410001, Nigeria; 4 School of Health and Life Sciences, Teesside University, Middlesbrough TS1 3BX, UK; 5 Department of Microbiology, Federal University Otuoke, Otuoke 562103, Nigeria; 6 Department of Microbiology, Imo State University, Owerri 460222, Nigeria; 7 Microbiology Research Unit, School of Human Sciences, London Metropolitan University, 166-220 Holloway Road, London N7 8DB, UK; 8 Department of Public Health Sciences, National Open University of Nigeria, Abuja 900108, Nigeria; 9 School of Molecular Bioscience, University of Glasgow, Glasgow G12 8QQ, UK

**Keywords:** antimicrobial resistance, One Health approach, antibiotic stewardship, surveillance systems, public health, animal husbandry, global action plan

## Abstract

Antimicrobial resistance (AMR) is a serious global health issue, aggravated by antibiotic overuse and misuse in human medicine, animal care, and agriculture. This study looks at the different mechanisms that drive AMR, such as environmental contamination, horizontal gene transfer, and selective pressure, as well as the severe implications of AMR for human and animal health. This study demonstrates the need for concerted efforts across the scientific, healthcare, agricultural, and policy sectors to control the emergence of AMR. Some crucial strategies discussed include developing antimicrobial stewardship (AMS) programs, encouraging targeted narrow-spectrum antibiotic use, and emphasizing the significance of strict regulatory frameworks and surveillance systems, like the Global Antimicrobial Resistance and Use Surveillance System (GLASS) and the Access, Watch, and Reserve (AWaRe) classification. This study also emphasizes the need for national and international action plans in combating AMR and promotes the One Health strategy, which unifies environmental, animal, and human health. This study concludes that preventing the spread of AMR and maintaining the effectiveness of antibiotics for future generations requires a comprehensive, multidisciplinary, and internationally coordinated strategy.

## 1. Introduction

Antimicrobial resistance (AMR) is the capacity of microorganisms including bacteria, fungi, viruses and parasites to change over time and withstand the effects of antibiotics that were originally effective against them [1]. AMR has become an increasing and severe global health challenge. Microorganisms can gain this resistance through various processes, including spontaneous mutations in their genetic makeup; these mutations modify the antibiotics’ target sites, thus making the antibiotics ineffective against the microorganism [2]. Additionally, microorganisms can acquire genes that are resistant to antibiotics from other organisms through horizontal gene transfer (HGT) processes including conjugation, transformation, and transduction [3]. AMR is exacerbated because the system works in a way that promotes the multiplication of microorganisms and increases their ability to withstand antimicrobial effects and the ability of resistant microorganisms to confer antimicrobial resistance to non-resistant microorganisms by the transfer of resistant genes.

The consequences of AMR are far-reaching and the treatment of infectious diseases has been severely hampered by AMR, prolonging illnesses and increasing healthcare costs. The rise in extensively drug-resistant (XDR) and multidrug-resistant (MDR) strains has made it increasingly difficult to treat common infectious diseases [4,5]. For example, the treatment of diseases like gonorrhea, tuberculosis, and other hospital-acquired infections has been made difficult by resistant bacteria strains [6,7]. The significant negative impact of AMR on healthcare means that more expensive and toxic pharmaceuticals are needed for treatments; it also means longer hospital stays for patients and demands for more intensive care. All these factors add up to the increasing overall costs of healthcare. For example, a study by the Rollins School of Public Health and Saint Louis University found that antibiotic resistance added USD 1383 per patient to treatment costs in the U.S., totaling around USD 2.2 billion nationally in 2014 [8]. AMR also has a significant impact on economies; it has been reported that in the absence of effective treatment interventions, AMR could reduce global gross domestic product (GDP) by 2–3.5% and force about 24 million people into extreme poverty globally by 2030 [9,10].

Furthermore, AMR is a factor in increased mortality. Infections from resistant strains have been reported to present with higher rates of morbidity and mortality, when compared to infections generated by non-resistant strains [11]. As of 2019, 1.27 million annual deaths globally were attributed to AMR; this number is expected to keep rising if the present trends continue [12]. The World Health Organization [13] has reported AMR in the top ten global public health threats to humanity, this highlights the crucial need for concerted global action to address AMR. Strong surveillance, careful administration and use of available antibiotics, and the development of new antimicrobials are very important to stay ahead in the continuous fight against AMR.

The study of AMR in the environment is very significant. The environment, including the soil, water, and food production systems, is a reservoir and conduit through which resistance genes are propagated [14,15]. Antibiotics and other antimicrobial compounds are regularly introduced into the environment through industrial processes, agricultural activities, and poor disposal of different pharmaceuticals [16,17]. These antimicrobial agents persist in the environment, exerting selective pressure on the microbial populations, in addition to encouraging the establishment and spread of resistant strains [18]. Furthermore, resistant bacteria and genes can spread from these environmental reservoirs to humans through different pathways, including food consumption, water use, and direct contact, posing considerable public health implications [19].

This paper aims to provide a comprehensive review of the environmental drivers of antimicrobial resistance (AMR), focusing on the mechanisms by which resistance genes spread in soil, aquatic systems, and food production environments. By critically analyzing the pathways through which AMR impacts food safety and public health, this paper highlights the complex interconnection between environmental contamination, human health, and agricultural practices. This study further evaluates the effectiveness of current surveillance systems, regulatory frameworks, and the One Health approach in mitigating AMR, while identifying gaps and proposing innovative strategies for reducing AMR at the global scale.

## 2. Development and Spread of AMR in Environmental Niches

### 2.1. Soil Environments

Antibiotics resistance genes (ARGs) are found in the complex and dynamic soil environment, which plays a major role in the emergence and spread of AMR. Agriculture, especially the widespread use of antibiotics in the cultivation of crops and livestock, is one of the major sources of antimicrobial compounds in the soil [20]. The use of animal waste as fertilizer introduces significant quantities of antimicrobials into soil ecosystems, influencing both microbial communities and their functional dynamics. This waste often contains residual antibiotics and other antimicrobial compounds that have not been entirely metabolized [21]. Antibiotics are commonly given to cattle for growth-promoting and medicinal purposes, which causes them to build up in animal waste, which is commonly used as fertilizer [22]. By adding large amounts of antimicrobial substances to the soil, this creates a selection pressure that encourages the growth of resistant microorganisms, raising important considerations for environmental health and agricultural practices. Research has shown that soils treated with manure from livestock raised on antibiotics exhibit significant changes in microbial composition, particularly a heightened prevalence of antibiotic-resistant bacteria when compared to untreated soils [23]. This increase in resistance genes within soil microbial communities carries potential risks for the transfer of ARGs to both plant-associated and human-associated microbes. This underscores the importance of implementing stringent management practices regarding the application of animal waste.

The problem of resistance development in soil microbial communities is further exacerbated by the use of sewage sludge, with resistant bacteria from human waste and antibiotic residues [24]. Resistant bacteria and residual antibiotics can remain in soil for extended durations, facilitating opportunities for HGT and augmenting the reservoir of ARGs in agricultural settings. Consequently, soils that receive sewage sludge demonstrate increased resistance levels, which may impact resistance patterns in microbiomes associated with crops and potentially enter the human food chain.

The diversity of the mechanisms underlying the development of resistance in soil bacteria is indicative of the complexity of soil as an ecological niche. Resistance genes are spread among soil bacteria through HGT. The HGT process of transformation entails taking up free DNA from the surroundings, which could be made easier by DNA adhering to soil constituents like clay minerals [25]. Studies have demonstrated that plasmids adsorbed onto minerals, such as montmorillonite and kaolinite, can withstand degradation and are available for uptake by competent bacteria, hence facilitating the dissemination of resistance genes [26,27]. Conjugation, the direct transfer of genetic material between bacteria, is also affected by soil components. For example, at low concentrations, certain minerals, such as goethite, encourage the conjugative transfer of resistance genes by damaging cell membranes, increasing bacterial interactions and gene transfer rates [28,29]. In transduction, the exchange of bacterial genetic material is facilitated by bacteriophages—viruses that infect bacteria. During transduction, bacteriophages incorporate pieces of the host bacterial DNA, including AMR genes, and transfer these genes to new bacterial hosts when they attach and infect them. Transduction is relevant in soil ecosystems, where a diversity of bacteriophages thrive. The interactions between bacteriophages and bacteria promote the transfer of resistance genes in the soil, enhancing the spread of AMR and the genetic diversity of microbial communities [30].

In addition to HGT, AMR development is significantly influenced by soil bacteria’s intrinsic resistance mechanisms. Soil bacteria inherently produce antibiotics as a competitive strategy. They have also developed a variety of resistance mechanisms to shield themselves from the antimicrobial substances produced by other microbes and their own antimicrobial compounds. A few of these methods are the enzymatic breakdown of antibiotics, the alteration of antibiotic targets, and efflux pumps, which remove antibiotics from cells [31]. Both naturally occurring and artificially produced antibiotics can select for bacteria that have these resistance characteristics in the soil environment, enriching the resistant populations. AMR also spreads quickly between different bacterial species and genera due to the high density and diversity of microbial communities in soil, which offer plenty of chances for the transfer of resistance genes.

The growth and dissemination of AMR can also be influenced by the physicochemical characteristics of soil, including pH, organic matter concentration, and the presence of metals and minerals [32]. Antibiotic bioavailability and environmental persistence may be impacted by interactions between soil components and antibiotics. To prolong their selective impact on bacterial communities, antibiotics can, for example, adsorb onto soil particles and be shielded from destruction. On the other hand, some elements of soil might decrease the effectiveness or inactivate antibiotics, which could result in exposure that is sub-lethal, thus resulting in the emergence of resistance [33]. Furthermore, resistance gene expression can be altered by the interactions between bacteria and soil components. As an example, research has demonstrated that specific minerals found in soil can cause oxidative stress in bacteria, resulting in the SOS response (a network of genes in bacteria that addresses DNA damage), which is known to facilitate the horizontal transfer of resistance genes [25,34].

The ecological and public health ramifications of AMR within soil ecosystems are of considerable importance. A comprehensive understanding of AMR in soil is vital for both agricultural productivity and human health, as soil acts as a significant reservoir for ARGs that can transfer to pathogens affecting plants, animals, and humans. In agricultural contexts, the prevalence of ARGs in the soil may influence the microbial communities associated with crops, potentially leading to increased plant diseases or diminishing the effectiveness of biocontrol agents. From a public health perspective, there exists a risk that ARGs present in soil could be conveyed to human-associated pathogens, either through direct human contact with soil or via the consumption of crops cultivated in contaminated soils [35]. Furthermore, the presence of AMR in soil represents a threat to broader ecosystems, as ARGs can migrate into adjacent water bodies through runoff, thereby facilitating the dissemination of resistance within the environment and possibly affecting human populations through water supply systems [36]. Consequently, it is essential to address AMR in soil through the implementation of sustainable agricultural practices and effective waste management strategies to curtail the spread of resistance.

### 2.2. Aquatic Environments

**Figure 1:**
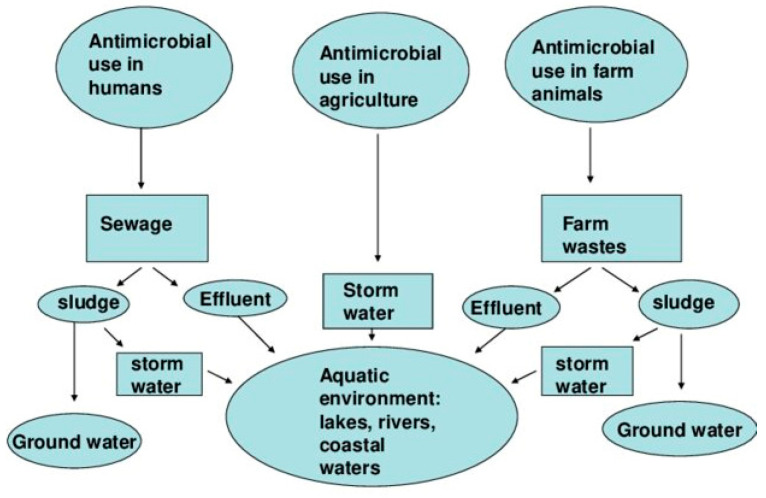
Pathways for the spread of antimicrobial residues and resistant bacteria in the aquatic environment [56].

Aquatic environments, which include lakes, rivers, oceans, and other bodies of water, are important in preserving ecological balance, promoting biodiversity, and providing resources for both animal and human populations. However, these natural environments are becoming increasingly contaminated because of anthropogenic activities involving the widespread use of antibiotics and their improper disposal. Municipal water ecosystems, notably those running through densely inhabited areas, act as major conduits for antibiotics into the environment. Antibiotic residues are accumulating in water bodies worldwide due to a variety of human activities, wastewater treatment facilities, agricultural runoff, and hospital system effluents (Figure 1) [37]. These pollutants persist in the environment, encouraging the emergence and spread of AMR and so fueling the increasing public health emergency. The implications of AMR in aquatic environments are substantial, as resistant pathogens have the potential to spread across international boundaries. This phenomenon has prompted global health initiatives, including the World Health Organization’s Global Action Plan on AMR, to address the challenge. Consequently, addressing AMR in aquatic ecosystems is not only crucial for safeguarding marine and freshwater environments but also aligns with overarching health and environmental policies, emphasizing its critical role in the preservation of both ecological integrity and public health. These pollutants include antibiotics used in aquaculture, such as oxytetracycline, florfenicol, and ormetoprim [38,39].

Antibiotics are not fully metabolized by humans and animals and are released in the aquatic environment through feces and urine, thereby contaminating the aquatic environment. According to Shigei et al. [40], urban rivers and other bodies of water are further contaminated by the resulting pharmaceutical pollutants, including paracetamol, ofloxacin, carbamazepine etc., which are frequently present in wastewater treatment plant effluents. Additionally, surface runoff from farms is important in the introduction of antibiotics and other pollutants into aquatic systems. These settings accelerate the selection and spread of resistance traits among microbial communities due to the presence of antimicrobial residues, antimicrobial-resistant bacteria (ARB), and ARGs, posing significant threats to the environment and public health [41]. Antibiotic pollution in aquatic ecosystems can disrupt the ecological balance, affecting native species and vital ecosystem functions. The presence of antibiotic-resistant bacteria can alter these environments’ microbiomes, negatively influencing nutrient cycling, primary production, and species survival, ultimately affecting biodiversity and ecosystem stability.

In aquatic environments, antibiotics and their residues, like those in soil, create selection pressure that favors the survival and growth of resistant bacteria. AMR is a global problem that is thought to have substantial reservoirs and transmission channels in certain ecosystems. Given that microorganisms are genetically adaptable, they may quickly adapt to the presence of antibiotics through processes like mutation, conjugation, transformation, and transduction [42]. This genetic plasticity drives the development of antimicrobial resistance in aquatic microbiomes. It is difficult to determine acceptable amounts of antibiotic residues in the environment because research indicates that even modest quantities of antibiotics in water might cause the selection of ARGs [43]. This problem is made worse because ARGs and ARBs cannot be completely removed from water by current water treatment processes, and this permits them to persist and spread across the environment [44,45]. Strategies to mitigate antibiotic pollution could involve advanced treatment techniques, such as membrane filtration and bioremediation, which aim to degrade antibiotics and ARGs more effectively. Additionally, optimizing antibiotic use in aquaculture and employing biosecurity measures can further reduce contamination in these environments.

ARG spread in aquatic environments is facilitated by different factors, including the direct discharge of untreated or poorly treated effluents from agricultural runoff, wastewater treatment plants, and the use of domestic wastewater for irrigation [46]. These processes deliver ARBs and ARGs to water bodies, from where they can be passed on to other organisms. This transfer happens via HGT, frequently resulting in the establishment of new, more resistant bacterial strains [47]. The prevalence of ARGs in aquatic settings highlights the complexities of the AMR problem, as these genes are difficult to degrade and can spread across multiple environments, including soil, water, sediments, and sewage [48].

Aquaculture, which makes a substantial contribution to global food production, is another important cause of antibiotic contamination in aquatic environments. The increasing number of ARGs in aquaculture systems is partly attributed to the use of antibiotics in the treatment and prevention of diseases in farmed fish [49]. Aquatic organisms can spread human pathogens through a variety of routes, such as eating contaminated fish or being exposed to the environment directly. Many of the antibiotics used in aquaculture—including tetracyclines, aminoglycosides, and macrolides—are vital to human medicine [50]. Numerous studies have reported on the indirect transfer of ARGs from aquatic habitats to human diseases. These findings underscore the significant risks to public health that arise from the use of antibiotics in aquaculture [45,51]. In response to these challenges, the implementation of sustainable aquaculture practices, including the use of probiotics, vaccination protocols, and enhanced husbandry techniques, may significantly diminish the dependence on antibiotics. This, in turn, could aid in curtailing the emergence and dissemination of antibiotic resistance.

Freshwater systems, such as rivers, lakes, and streams, are particularly susceptible to antibiotic contamination given their proximity to anthropogenic activities. These systems are continually inundated with antibiotics, ARBs, and ARGs from sources including wastewater treatment facilities, chemical production plants, and agricultural wastewater [52]. The presence of ARBs in these ecosystems has been reported even in remote and extreme locations, such as Eastern Siberia’s permafrost and Antarctic freshwater lakes, implying that AMR spread is a global concern that goes beyond geographical boundaries [53].

Antibiotic pollution has a substantial effect on marine environments even though it differs from freshwater systems in the processes of AMR’s development and transmission. ARBs from terrestrial sources are frequently carried by coastal runoff, which adds to the build-up of ARGs in marine environments [45]. The selection pressure for resistant bacteria is further increased by directly releasing antibiotic residues from operations like aquaculture into marine habitats. According to Miranda et al. [54], there is evidence that Chilean marine salmonid farms utilize a significant amount of antibiotics, which may be a contributing factor to the emergence of antibiotic-resistant marine bacteria. Given that humans become exposed to marine ARGs through activities like swimming, fishing, and seafood consumption, the possibility of a bidirectional transfer of ARGs between human populations and marine environments cannot be ruled out [55].

### 2.3. Food Production Systems

The development of AMR and the application of antimicrobials in food production are closely linked. Subtherapeutic doses of antibiotics are frequently given to animals to enhance their growth and prevent diseases; this practice provides an ideal environment for the emergence of resistance strains. In the animal’s gastrointestinal tract, these resistant bacteria can proliferate, persist, and can also pick up extra resistance genes through HGT, increasing their ability to withstand antibiotic treatments [57]. Cross-resistance emerges because the antibiotics used in human medicine are identical to those used in animals. As a result, these resistant bacteria represent a serious threat to public health when they infiltrate the environment or the food chain since they can result in diseases that are difficult to treat.

Similar to the emergence of AMR in the soil, one major factor contributing to AMR in food production systems is the extensive use of antibiotics in livestock husbandry and agriculture [58]. The magnitude of this problem is demonstrated by the fact that the amount of antibiotics employed in agriculture often exceeds the amount used in human treatment in several countries [59]. Introducing antibiotics to animal feed is a typical technique in intensive farming systems, where housing so many animals in close quarters increases the danger of disease transmission. The regular use of antibiotics in this way, however, is questionable since it contributes significantly to the development and spread of resistant bacteria that might infect humans. There are multiple routes via which resistance from animals can reach humans and promote AMR. Consuming animal products like meat, milk, or eggs that contain resistant bacteria is one of the primary routes [60]. These bacteria can survive cooking, especially if food is not properly cooked, and they can infect people with illnesses that are resistant to treatments. Direct interaction with animals or their natural environments is an important additional pathway, especially for those in the agricultural or animal husbandry businesses. In these environments, humans can come into close contact with animals or inhale dust particles, which can spread resistant bacteria [61].

**Figure 2:**
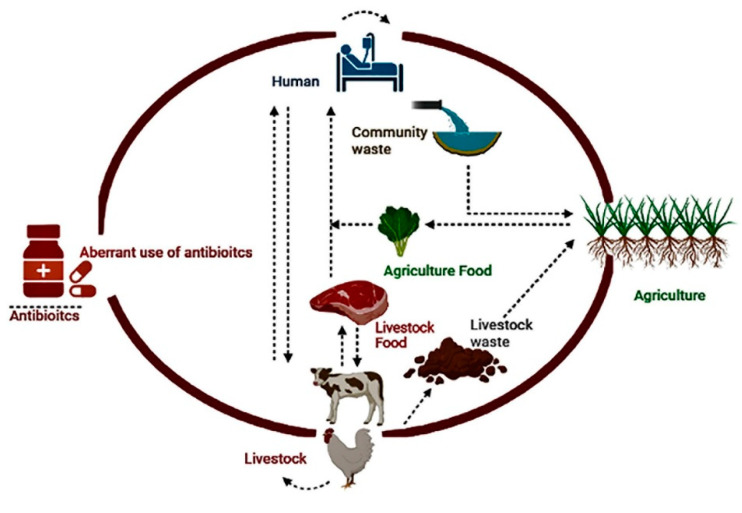
Pathways of antibiotics contamination of soil, water, and food and how they spread in animals and humans [63].

Antimicrobial usage in food production is a complex topic that includes preventing and controlling the growth of bacteria in food items in addition to treating animal diseases. As already stated, antimicrobials are employed not just in livestock but also in aquaculture, agriculture, and other fields, which expands the application of AMR research. Antibiotics can be added to the surrounding water and cause the selection of bacteria that are resistant in aquatic environments. Antibiotics are used in aquaculture for the prevention and treatment of bacterial infections in fish. Similarly, using antimicrobials in crop production can contaminate soil and water, which can lead to the spread of resistant bacteria. These actions have the combined effect of widely disseminating resistant bacteria throughout various ecosystems (Figure 2) which may eventually make their way into human populations through environmental exposure, water consumption, or food consumption [62].

## 3. Implications of Environmental Antimicrobial Resistance on Food Safety

### 3.1. Transmission of Resistant Bacteria Through Food Chains

AMR bacteria and resistance genes can contaminate food in several ways which include cross-contamination during food handling, intentionally introducing bacteria such as probiotics or other bioconserving microorganisms which may carry resistance genes during food processing, and the use of antibiotics in agriculture [64]. Although bioconserving microorganisms which primarily are used to preserve and extend the shelf life of food products by inhibiting the growth of spoilage organisms and pathogens are of benefit to the food processing industry, they can pose a threat, especially in raw or minimally processed foods, which carry a higher risk because they can include live, resistant bacteria that, when consumed, might pass resistance genes to human bacteria [65]. Less processing can also keep stressed bacterial cells alive, which raises the possibility of resistance transfer even more.

#### 3.1.1. Contamination During Food Processing and Handling

Food handlers are often linked to the spread of AMR bacteria as a result of cross-contamination from handling contaminated food or inadequate hygiene procedures [66]. This can lead to the dissemination of AMR bacteria across different food products, potentially reaching many consumers through various food channels which poses a significant threat to public health. Once introduced into the food supply, AMR bacteria can persist in the environment and spread through various means, including agricultural runoff and waste disposal, exacerbating the problem of AMR by contaminating additional food sources and contributing to the broader spread of resistance [67]. With the increase in demand for raw or minimally processed food, the chances for the proliferation of AMR bacteria have also increased as these foods, such as fresh fruits, vegetables and raw milk, may contain live, unstressed bacterial cells and are usually consumed without first going through any processing or preservation procedures [64]. This heightens the risk of AMR bacteria proliferation in the human population, making it more difficult to treat bacterial infections with conventional antibiotics and potentially leading to increased health risks and challenges in managing resistant infections.

Usually, different food processing and preservation methods are applied to extend the shelf life of food or improve its taste or nutritional quality. However, these processes might affect bacterial flora differently, with some bacteria surviving these techniques without inhibited growth, while others may be inhibited, resulting in stressed or sub-lethally damaged cells [64,68]. Some of these bacteria that may survive these processes and continue to grow, may potentially include AMR bacteria, thereby contributing to the spread of resistance. Le Devendec et al. [69] evaluated the impact of heat treatment on AMR *E. coli* and found that they survived after a 60 °C incubation for 5 to 10 min while retaining their resistance genes. Although theoretically AMR genes surviving heat treatment and stomach acid could potentially transfer to other gut microbiota and contribute to AMR, there is currently no evidence in the literature showing that AMR genes from heat-treated or cooked food transfer to gut microbiota [70]. Non-thermal food processing methods like high pressure and ionizing radiation help to improve food safety by damaging microbial cell membranes without affecting nutritional value or taste; however, these techniques might release genetic material related to AMR, potentially transferring it to environments or bacteria that come into contact with the treated food, thereby exacerbating the spread of AMR within the food chain [66,71]. A study by Zarzecka et al. [68] examined how high-pressure processing affects antibiotic resistance in commercial starter culture strains. Results from the study found that high pressure reduced the expression of aminoglycoside resistance genes but increased the expression of tetracycline and chloramphenicol resistance genes, with variations depending on the pressure level applied, potentially leading to the spread of different resistance genes through the food chain.

#### 3.1.2. Intentionally Introducing Microorganisms into Food as Supplementary Substances

The intentional introduction of microorganisms into food is a common practice in the food industry, usually aimed at enhancing food quality, safety, and nutritional value [72]. These microorganisms, often added as probiotics, bacteriophages, starter cultures, or biopreservatives, play crucial roles in fermentation, flavor development, and food preservation [64]. However, this practice can also contribute to the transmission of AMR within the food chain as these microorganisms may carry ARGs which can be transferred to other bacteria within the food chain through different mechanisms, such as horizontal gene transfer, conjugation, or selective pressure [64,73].

In various food processing contexts, supplementary substances such as growth promoters, preservatives, or additives can influence the microbial environment and potentially facilitate the transfer of AMR genes [74]. These substances might create conditions that either support the survival of resistant bacteria or promote their ability to exchange genetic material. For instance, substances like antibiotics used as preservatives or growth promoters in animal feed can exert selective pressure, allowing resistant bacteria to thrive and potentially transfer resistance genes through mechanisms like conjugation or horizontal gene transfer [75]. Similarly, certain preservatives or additives might affect the microbial community in food products in ways that enhance the survival of resistant strains, thus increasing the likelihood of gene transfer [74]. This was demonstrated in a study [76] where ampicillin resistance was transferred from a donor strain of *Salmonella* Typhimurium DT104 to a recipient strain of *Escherichia coli* K12. Similarly, Toomey et al. [77] demonstrated that lactic acid bacteria (LAB) such as *Enterococcus faecalis* and *Lactococcus lactis* can transfer resistance to pathogenic strains like *Listeria*, *Salmonella*, *Staphylococcus aureus*, and *E. coli* in fermented milk, emphasizing LAB’s potential role as a source of resistance determinants. This suggests that while LAB is traditionally considered beneficial, its potential contribution to the spread of AMR poses a serious risk to public health, as resistant strains of pathogenic bacteria could emerge and proliferate, which threatens the safety of food products.

Most LAB colonize the intestines briefly, but probiotics can attach to intestinal epithelial cells, allowing them to colonize longer, thereby increasing the risk of horizontal gene transfer of ARGs from these LAB to other gut microbiota, including pathogenic bacteria [78]. LAB, including species from *Lactobacillus*, *Lactococcus*, *Leuconostoc*, and *Pediococcus*, are widely used as probiotics and starter cultures due to their established safety [79]. However, despite their safety record, numerous *Lactobacillus* species have been reported to possess antibiotic resistance, both intrinsic and acquired, to various antibiotics such as aminoglycosides, tetracycline, and erythromycin [80,81,82]. Foods containing probiotics are widely consumed, and microorganisms present in large numbers in food or human intestines are more likely to transfer AMR genes. This risk is particularly relevant for probiotics and biopreservatives added in substantial amounts to food products, as these microorganisms can harbor and potentially transfer resistance genes to other bacteria in the gut [79]. Additionally, the starter cultures used in fermentation processes, which often proliferate extensively, can act as reservoirs for AMR genes, further amplifying the potential for gene transfer within the food chain [80]. The widespread consumption of such foods could inadvertently contribute to the dissemination of resistance genes, posing challenges for public health. Similarly, bacteriophages have been implicated as environmental reservoirs of resistance genes, although they can be employed to render foodborne pathogens and spoilage organisms inactive [64]. In their study, Colomer-Lluch et al. [83] investigated the occurrence of three β-lactam ARGs (*blaTEM*, *blaCTX-M9*, and *mecA*) in bacteriophage DNA from environmental water samples. Using quantitative PCR, these genes were found in all tested samples, with high gene copy numbers correlating with fecal pollution levels, except for *mecA*, which was more abundant in river water, suggesting that bacteriophages can act as reservoirs of AMR genes, facilitating the spread of resistance within the food chain through environmental contamination.

### 3.2. Risk Assessment of Resistant Bacteria in Food Products

Several health implications are associated with the presence of AMR bacteria in food products. According to estimates from the Centres for Disease Control and Prevention (CDC), over 35,000 people die from AMR-related infections every year in the United States alone, where at least 2.8 million individuals contract infections [84]. Infections resistant to antibiotics are also linked to higher rates of complications and mortality, longer hospital stays, and higher healthcare expenses. The prevalence of AMR bacteria in food products poses a significant public health risk, necessitating comprehensive risk assessment to mitigate potential outbreaks and protect consumer health [85]. Public health organizations, including the CDC, Food and Drug Administration (FDA), and World Health Organization WHO, are integral to the monitoring and intervention strategies related to AMR. Their initiatives encompass the surveillance of AMR trends, the formulation of guidelines aimed at reducing antibiotic usage in agricultural practices, and the encouragement of research focused on the pathways of AMR transmission. These agencies are pivotal in the orchestration of risk assessment frameworks that facilitate the monitoring of AMR throughout the food supply chain, thereby enabling the implementation of effective preventative measures.

For dairy cattle, there is limited information on the risk of AMR transmission to humans, but the main concern is the development of resistance in livestock gut bacteria and the potential transfer of AMR genes to zoonotic pathogens that can infect humans [86]. Several studies have investigated the role of food as an important vehicle in the transmission of resistant bacteria to the digestive tract of consumers. For instance, in a cross-sectional study by Beyene et al. [87], involving 193 samples from dairy farms and abattoirs, *Staphylococcus* species were isolated from 47.7% of the samples, with a higher prevalence in dairy farms (50%) compared to abattoirs (46.3%). Among the isolates, 95.3% exhibited resistance to penicillin-G, while 88.4% showed resistance to nalidixic acid. Notably, significant multidrug resistance was observed, particularly in *S. aureus*, where 73.3% were resistant to vancomycin, and all isolates displayed resistance to more than three drugs. A similar study conducted in Japan by Hiroi et al. [88] found that *Campylobacter coli* had a higher resistance compared to *Campylobacter jejuni* in retail meats and food-animal feces. Additionally, methicillin-resistant *Staphylococcus aureus* (MRSA) was identified in 3% of meat samples, with no vancomycin-resistant enterococci detected. *S. aureus* showed high resistance to ampicillin and tetracycline, and identical resistance patterns were observed in *C. jejuni* from both a hospital patient and chicken samples, suggesting a potential link between the meat and the infections.

As meat consumption has risen, so has the demand for meat products due to their desirable sensory properties and efficient use of carcass parts unsuitable for fresh consumption [89]. The pork industry, in particular, has expanded significantly due to pork’s high-quality, low-cost protein, even though pigs have become major reservoirs of AMR due to the misuse of antibiotics in their production process for the growth promotion or prophylactic treatment of diseases [90]. Bouchami et al. [91] carried out a study to determine if the pork production chain is a source of MRSA ST398 for human colonization and infection by analyzing samples from live pigs, meat, the environment, and slaughterhouse workers. They found that MRSA ST398, which is multidrug-resistant and carries genes for biocide resistance and enterotoxins, was present along the entire pork production chain and in workers, with evidence of cross-transmission between live pigs, meat, and workers. Similarly, Cao et al. [92] used samples from 21 volunteers as well as pig and poultry carcasses to confirm the hypothesis that foodborne bacteria transfer AMR genes to the human gut microbiota. They found that AMR genes that were resistant to several antimicrobial classes, including vancomycin, tetracycline, and macrolides, were present in both humans and food animals. To address this growing threat, a comprehensive risk assessment and mitigation approach is essential. Risk mitigation strategies play a pivotal role in controlling the spread of AMR within the meat industry. Adopting best practices necessitates the implementation of more stringent regulations regarding antibiotic usage, restricting its application solely to therapeutic purposes. Moreover, the enforcement of residue testing is vital to ensure compliance with these regulations. The establishment of comprehensive biosecurity measures, including the isolation of infected animals and the enhancement of hygiene standards within slaughterhouses, is critical for minimizing the risk of cross-contamination. Furthermore, augmented training for workers on safe handling and processing practices can significantly mitigate transmission risks, thereby protecting both employees and consumers alike. Surveillance and monitoring should be prioritized to track AMR bacteria throughout the meat production chain. Regular sampling and genetic analysis can help identify specific resistance genes and their potential for transfer between animals, food products, and humans; this is crucial for understanding the scope of the problem and implementing targeted interventions. Additionally, regulations must be enforced to limit the use of antibiotics in the meat production process, coupled with improved biosecurity measures and hygiene practices in slaughterhouses and processing plants to minimize the risk of cross-contamination. Public health interventions should focus on educating both workers and consumers about the risks associated with AMR and promoting safe handling, cooking, and consumption practices.

#### Outbreaks of Foodborne Illnesses Caused by Resistant Bacteria, Europe as a Case Study

The 2011 foodborne disease outbreak in Central Europe, particularly Germany, due to a highly resistant hybrid strain of O104:H4 STEC from contaminated sprouts serves as an important example of the threat AMR poses to the food and health sectors. This outbreak resulted in nearly 4000 infections, with over 900 cases of hemolytic uremic syndrome (HUS) and 54 deaths [93]. The strain was notable for its combination of genetic traits from Enteroaggregative *Escherichia coli* (EAEC) and its ability to produce Shiga toxin (*stx2a*), despite lacking other traditional STEC virulence markers like *eae* and *hlyA* [94]. Crucially, the strain also carried ARGs *bla*_CTX-M-15_ and *bla*_TEM-1_ on plasmids, which contributed to the difficulty in treating infections. The presence of these resistance genes heightened the severity of the outbreak, complicating treatment options and exacerbating the public health crisis [95]. This highlighted the need for increased vigilance regarding AMR spread from food.

In 2018–2019, the European Food Safety Authority (EFSA) and European Centre for Disease Prevention and Control (ECDC) jointly analyzed data from 28 EU Member States on AMR in zoonotic bacteria including *Salmonella* and *Campylobacter*, as well as indicator bacteria such as *Escherichia coli* and MRSA from humans, animals, and food [96]. In 2018, high levels of resistance were reported among human *Salmonella* isolates, with sulfonamides (30.5%), tetracyclines (28.8%), and ampicillin (25.9%) showing significant resistance. Resistance varied widely by serovar and country, with monophasic *S.* Typhimurium and *S.* Kentucky showing extremely high resistance, while *S.* Enteritidis showed lower resistance overall. Notable variations included higher resistance to ampicillin in Italy for *S.* Infantis and lower resistance in Malta for monophasic *S.* Typhimurium. Gentamicin resistance was generally low except for in *S.* Kentucky, and chloramphenicol resistance was low overall but moderate in *S.* Typhimurium. *Salmonella* spps. from human cases in 2019 are highly resistant to ampicillin, sulfonamides, and tetracyclines but have low resistance to third-generation cephalosporins like cefotaxime (1.8%) and ceftazimide (1.2%) [97]. These high resistance levels, especially to critical antibiotics like sulfonamides and tetracyclines, complicate treatment for patients infected with these resistant strains of *Salmonella* and other bacteria, leading to longer illness durations, increased healthcare costs, and higher morbidity and mortality rates. Following this trend, a more concerted global effort is needed to avert the “antibiotics catastrophe” that the world may face in the coming decades.

## 4. Public Health Impact and Human Health Risks of AMR

### 4.1. Global Landscape of AMR

The global landscape of AMR is one of increasing incidence and the burden of multidrug-resistant infections that are spreading out of control. AMR is the cause of 4.95 million deaths worldwide, with 1.27 million deaths directly related to resistant pathogens (including *Escherichia coli*, *Staphylococcus aureus*, and *Klebsiella pneumoniae*), which are among the most alarming, according to the WHO priorities [98]. These organisms are resistant to many types of antibiotics such as fluoroquinolones or β-lactams, which are considered the mainstay of treatment. Since their resistance occurs more frequently and easily than that of other antibiotics, they pose an even greater public health threat in low- and middle-income countries (LMICs), where healthcare infrastructure is weaker. For instance, mortality rates are dramatically increased, and treatment is more complicated, in regions with a high prevalence of infections such as sub-Saharan Africa and South Asia [99].

Reducing the burden of AMR involves infection prevention, vaccination, and encouraging the use of appropriate antibiotics, and centers on high-burden regions. The spread of resistant bacteria from contaminated wastewater is another environmental culprit. The economic impact is immense, with AMR-related health expenditures and productivity losses exceeding USD 55 billion in the US [67]. AMR is a global problem, and reducing its burden requires coordinated efforts across the globe in increased surveillance, new drug development, and public health interventions to prevent this growing threat from worsening.

Table 1 provides an overview of the global landscape of AMR, summarizing key studies, statistics, and initiatives across various aspects of AMR. It includes details on the global burden and mortality caused by drug-resistant infections, highlighting the critical pathogens involved, such as *E. coli*, *S. aureus* and *K. pneumoniae*. The table also explores the impact of resistance to specific antibiotics, regional disparities in AMR burden, and the challenges faced in different healthcare settings and populations.

### 4.2. Public Health Impact of AMR

The public health impact of AMR is profound, affecting not only individual patients but also healthcare systems and societies at large.

#### 4.2.1. Increased Morbidity and Mortality

AMR significantly contributes to higher morbidity and mortality rates worldwide. Infections caused by resistant pathogens are more difficult to treat, often requiring longer hospital stays and more intensive care. Pneumonia, tuberculosis, and sepsis—diseases that were once easily treated with antibiotics—now often result in death as bacteria grow increasingly resistant to drugs [114]. Over time, this threatens to reverse much of the progress medicine has made over the past century, turning even ordinary infections into potentially serious conditions. In the absence of antibiotics, even a minor infection could kill us, highlighting the need for new antimicrobial strategies worldwide.

#### 4.2.2. Complications in Medical Procedures

AMR complicates a wide range of medical procedures and treatments. Modern medicine relies heavily on effective antibiotics for surgeries, cancer therapy, organ transplants, and the care of premature infants. The success of these procedures is threatened by the growing prevalence of resistant infections. For instance, patients undergoing chemotherapy or major surgery are at higher risk of infection, and without effective antibiotics, their chances of recovery diminish significantly [115].

#### 4.2.3. Economic Burden

The economic impact of AMR is substantial. We have earlier established that treating resistant infections are more expensive due to the need for more complex and prolonged treatment regimens, including the use of newer, more expensive antibiotics. Extended hospital stays also increase healthcare costs for both patients and health systems. For instance, resistant infections in U.S. hospitals in 2014 have been associated with an additional USD 1383–1488 in treatment costs per infection, and AMR-related hospitalizations are often twice as long as those for non-resistant infections [8]. This cost amounted to a national treatment cost of approximately USD 2.2 billion in that year. The World Bank predicts that by 2050, the global economy could lose up to USD 100 trillion due to AMR [115]. In the United States alone, AMR adds an estimated USD 20 billion in direct healthcare costs annually, with additional costs due to lost productivity as high as USD 35 billion [116]. Developing countries, where healthcare resources are already limited, face even greater challenges in managing the economic burden of AMR.

#### 4.2.4. Impact on Global Health Security

AMR poses a significant threat to global health security. The spread of resistant pathogens knows no borders, making it a global issue that requires coordinated international action. Outbreaks of resistant infections can lead to public health emergencies, as seen with multidrug-resistant tuberculosis (MDR-TB) and MRSA [117]. The emergence of “superbugs” that are resistant to multiple antibiotics further complicates the global health landscape. These pathogens can spread rapidly within communities and across countries, necessitating robust surveillance and response mechanisms to detect and contain outbreaks [118].

#### 4.2.5. Reduced Effectiveness of Antibiotics

The overuse and misuse of antibiotics in humans, animals, and agriculture have accelerated the development of resistance. As a result, common antibiotics are losing their effectiveness, leading to a situation where even minor infections could become untreatable. This “post-antibiotic era” could turn back the clock on medical advancements, making routine infections deadly once again [119].

#### 4.2.6. Impact on Vulnerable Populations

Certain populations are more vulnerable to the effects of AMR, including the elderly, young children, and immunocompromised individuals. These groups are more likely to require antibiotics and are at higher risk for resistant infections. In developing countries, where access to healthcare and antibiotics is often limited, the impact of AMR can be even more devastating, leading to higher mortality and morbidity rates [117].

### 4.3. Human Health Risks of AMR

#### Infections Caused by Resistant Bacteria and Challenges in Treatment and Increased Healthcare Costs

AMR can occur in various settings, including hospitals and communities, and through the consumption of contaminated food. Resistant bacteria can lead to severe illnesses such as urinary tract infections, respiratory infections, and bloodstream infections, among others. The presence of AMR in the environment ensures that these bacteria can persist and spread, increasing the likelihood of human exposure and causing subsequent infection [110].

Resistant bacteria, such as MRSA, ESBL-producing *E. coli*, and carbapenem-resistant *Pseudomonas aeruginosa*, complicate treatment efforts by limiting the effectiveness of standard antibiotics and often requiring the use of more expensive and less readily available alternatives. For example, respiratory infections caused by penicillin-resistant *Streptococcus pneumoniae* and multidrug-resistant *Mycobacterium tuberculosis* necessitate higher doses, longer treatment durations, and the use of second-line drugs, which are often more toxic and less effective [101].

The economic impact of treating infections caused by resistant bacteria is substantial. In addition to the need for more expensive medications and prolonged hospital stays, interventions such as intensive care and surgical procedures are also causes of increased hospital costs. For example, the treatment of MDR-TB and XDR-TB involves the long-term use of costly second-line antibiotics, significantly driving up overall healthcare expenses. Similarly, managing bloodstream infections caused by carbapenem-resistant *Klebsiella pneumoniae* requires extended ICU stays and high-cost antibiotics, contributing to increased treatment costs [106].

Table 2 outlines various infections caused by antibiotic-resistant bacteria, detailing the pathogens involved, types of resistance, and associated challenges and impacts. Respiratory infections, such as those caused by *Streptococcus pneumoniae*, *Mycobacterium tuberculosis*, *Haemophilus influenzae*, and *Pseudomonas aeruginosa*, show resistance to penicillin, multiple drugs, and carbapenems [120]. These infections result in increased morbidity and mortality, with limited treatment options and high costs. Urinary tract infections (UTIs) caused by pathogens like *Escherichia coli*, *Klebsiella pneumoniae*, *Proteus mirabilis*, and *Enterococcus faecalis* exhibit resistance to β-lactamase, carbapenems, and vancomycin, leading to high recurrence rates and complications, particularly in healthcare settings and vulnerable populations [120].

Skin and soft tissue infections, often due to *Staphylococcus aureus* (including MRSA), *Pseudomonas aeruginosa*, and *Acinetobacter baumannii*, are challenging to treat and have high transmission rates, increasing treatment costs [121]. Gastrointestinal infections from *Salmonella* spp., *Clostridium difficile*, *Shigella* spp., and *Campylobacter jejuni* show resistance to fluoroquinolones, multiple drugs, and macrolides, causing severe diarrhea, colitis, and significant healthcare burdens [122]. Bloodstream infections, involving pathogens like *Enterococcus faecium*, *Klebsiella pneumoniae*, *Staphylococcus aureus* (including MRSA), and *Acinetobacter baumannii*, present high mortality rates and prolonged hospital stays due to vancomycin, carbapenem, methicillin, and multidrug resistance [120].

Sexually transmitted infections (STIs) caused by *Neisseria gonorrhoeae* and *Treponema pallidum* demonstrate growing resistance to cephalosporins, azithromycin, and penicillin, which limit treatment options and poses public health risks. Hospital-acquired infections, with pathogens like *Pseudomonas aeruginosa*, *Enterobacter* spp., *Acinetobacter baumannii*, and *Escherichia coli*, are difficult to control and lead to extended hospital stays and increased healthcare costs due to carbapenem and multidrug resistance. Gastrointestinal infections from *Helicobacter pylori* and *Vibrio cholerae* exhibit resistance to clarithromycin and tetracycline, complicating treatment and increasing morbidity [123].

Central nervous system infections caused by *Neisseria meningitidis* and *Streptococcus pneumoniae* are resistant to penicillin and ciprofloxacin, resulting in high fatality rates and complex treatments. Lastly, bone and joint infections, typically due to *Staphylococcus aureus* (including MRSA) and *Pseudomonas aeruginosa*, require prolonged treatment and have high recurrence rates due to methicillin and carbapenem resistance. Overall, antibiotic-resistant infections across these categories significantly challenge healthcare systems, increase morbidity and mortality, and necessitate urgent action to mitigate their impacts [124].

**Table 2:** Infections caused by resistant bacteria, challenges in treatment, and increased healthcare costs.

Infection Category	Common Resistant Bacteria	Notable Antibiotic Resistance Types	Impact and Challenges
Respiratory Infections	*Streptococcus pneumonia*, *Mycobacterium tuberculosis*, *Haemophilus influenzae*, and *Pseudomonas aeruginosa*	Penicillin resistance, MDR-TB, XDR-TB, and β-lactam resistance, and carbapenem resistance	Increased morbidity and mortality, limited treatment options, and high treatment costs
Urinary Tract Infections	*Escherichia coli*, *Klebsiella pneumoniae*, *Proteus mirabilis*, and *Enterococcus faecalis*	ESBL-producing strains, carbapenem resistance, and vancomycin resistance	High recurrence rates, complicated infections in healthcare settings, and difficult management in vulnerable populations
Skin and Soft Tissue Infections	*Staphylococcus aureus (MRSA)*, *Pseudomonas aeruginosa*, and *Acinetobacter baumannii*	Methicillin resistance, carbapenem resistance, and multidrug resistance	Difficult to treat, high transmission rates in community and hospital settings, and increased treatment costs
Gastrointestinal Infections	*Salmonella* spp., *Clostridium difficile*, *Shigella* spp., and *Campylobacter jejuni*	Fluoroquinolone resistance, multidrug resistance, and macrolide resistance	Severe diarrhea and colitis, high relapse rates, and increased healthcare burden
Bloodstream Infections	*Enterococcus faecium*, *Klebsiella pneumoniae*, *Staphylococcus aureus (MRSA)*, and *Acinetobacter baumannii*	Vancomycin resistance, carbapenem resistance, methicillin resistance, and multidrug resistance	High mortality rates, challenges in infection control, and prolonged hospital stays
Sexually Transmitted Infections	*Neisseria gonorrhoeae* and *Treponema pallidum*	Resistance to cephalosporins, resistance to azithromycin, and resistance to penicillin	Increasing prevalence, limited treatment options, and public health implications
Hospital-acquired Infections	*Pseudomonas aeruginosa*, *Enterobacter* spp., *Acinetobacter baumannii*, and *Escherichia coli*	Carbapenem resistance, multidrug resistance, and ESBL-producing strains	Prolonged hospital stays, increased healthcare costs, and difficult to control outbreaks
Gastrointestinal Infections	*Helicobacter pylori* and *Vibrio cholera*	Clarithromycin resistance and tetracycline resistance	Complicated treatment and increased morbidity
Central Nervous System Infections	*Neisseria meningitidis* and *Streptococcus pneumonia*	Penicillin resistance and ciprofloxacin resistance	High fatality rates and complicated treatment
Bone and Joint Infections	*Staphylococcus aureus (MRSA)* and *Pseudomonas aeruginosa*	Methicillin resistance and carbapenem resistance	Prolonged treatment and high recurrence rates

Sources: [101,120,124].

### 4.4. Public Health Policies Addressing AMR

Public health policies and regulations play a pivotal role in controlling and mitigating the impact of AMR. These policies may include guidelines for the prudent use of antibiotics in both human and veterinary medicine, regulations on the discharge of pharmaceuticals and other contaminants into the environment, and standards for infection prevention and control in healthcare settings. Public health regulations also support the development and implementation of national and international action plans to combat AMR [125]. These plans often emphasize the importance of a One Health approach, which recognizes the interconnectedness of human, animal, and environmental health in addressing the AMR challenge.

#### 4.4.1. Antimicrobial Stewardship

o Regulations often include guidelines for the appropriate use of antibiotics to prevent overuse and misuse, which are primary drivers of resistance. This includes promoting the prescription of antibiotics only when necessary and ensuring the correct dosages and duration of treatments [126,127].
o Stewardship programs are implemented in healthcare settings to educate healthcare providers and patients about the responsible use of antimicrobials [128].

#### 4.4.2. Infection Prevention and Control

o Policies emphasize the importance of infection prevention and control measures in healthcare facilities, such as hygiene practices, sterilization procedures, and isolation protocols for patients with resistant infections [115,129].
o Vaccination programs are also promoted to reduce the incidence of infections that might otherwise require antibiotic treatment [130].

#### 4.4.3. Research and Development

o Public health regulations support research into new antibiotics, alternative treatments, and rapid diagnostic tools. This is critical for staying ahead of evolving resistant strains and ensuring that effective treatments remain available [131].
o Incentives for pharmaceutical companies to develop new antimicrobials and support for public-private partnerships are often part of these strategies [126].

#### 4.4.4. Education and Awareness

o Increasing awareness about AMR among healthcare professionals, policymakers, and the public is a key component of most AMR policies. Educational campaigns aim to inform about the dangers of AMR and the importance of prudent antimicrobial use [114,119].
o Training programs for healthcare providers ensure that they are equipped with the knowledge and tools to combat AMR effectively [128].

#### 4.4.5. Surveillance, Monitoring, and Reporting Systems

Surveillance and reporting systems for AMR are essential tools in the global fight against antibiotic-resistant infections. These systems provide crucial data on the prevalence, spread, and trends of resistant pathogens, enabling timely and informed public health responses. For instance, data collected through these systems have directly influenced policy decisions, such as the implementation of targeted antibiotic stewardship programs and the allocation of resources for infection control measures [106]. The WHO has established the Global Antimicrobial Resistance and Use Surveillance System (GLASS), which standardizes data collection and reporting across participating countries. The GLASS monitors resistance in human and veterinary pathogens, allowing for a comprehensive view of AMR trends and facilitating global comparisons [103]. In Europe, the European Antimicrobial Resistance Surveillance Network (EARS-Net) plays a pivotal role. It collects, analyses, and disseminates data on AMR from clinical samples across member countries. Looking forward, improvements in AMR surveillance could include enhanced international collaboration, such as joint data-sharing initiatives, and advancements in technology, like the integration of artificial intelligence for real-time data analysis. This network helps identify emerging resistance patterns and informs public health strategies at both national and international levels [102]. In the United States, the National Antimicrobial Resistance Monitoring System (NARMS) tracks changes in the susceptibility of intestinal bacteria found in ill people, retail meats, and food animals. This tripartite system, involving the CDC, FDA, and United States Department of Agriculture (USDA), provides critical information for risk assessments and policy decisions aimed at curbing AMR [132].

The Study for Monitoring Antimicrobial Resistance Trends (SMART) is another significant initiative. The SMART collects data through scientific and medical journals and public databases like EARS-Net. It focuses on temporal and spatial trends in AMR, offering valuable insights into local, national, and international resistance dynamics [12]. These surveillance systems are supported by diagnostic advancements, such as the development of commercial laboratory kits for phenotypic and molecular testing. These kits enable the rapid and accurate detection of resistant bacteria and resistance markers, enhancing the efficiency of surveillance efforts [108]. Public health campaigns, such as World Antimicrobial Awareness Week (WAAW), further bolster these efforts by raising awareness and promoting best practices in antibiotic use. These campaigns have been shown to significantly increase public awareness and encourage responsible antibiotic use, evidenced by measurable outcomes such as reduced antibiotic prescriptions in participating regions. Initiatives like the British Society for Antimicrobial Chemotherapy’s “Stop Superbugs” campaign and the CDC’s “Combating Antimicrobial Resistance” portfolio provide resources and guidance to support public awareness and health literacy [106].

Effective AMR policies prioritize robust surveillance systems to monitor the prevalence and spread of antimicrobial-resistant infections. This data collection helps in identifying trends, outbreaks, and high-risk areas, enabling timely and targeted interventions [84,117]. Monitoring systems are also essential for tracking antimicrobial use in both human and veterinary medicine, ensuring that usage patterns do not contribute to the development of resistance [133].

#### 4.4.6. Regulatory Frameworks

o Governments and international bodies establish regulatory frameworks to control the sale and distribution of antibiotics. This includes restricting over-the-counter sales of antibiotics and enforcing prescription-only policies [133,134].
o Regulations also encompass the agricultural sector, limiting the use of antibiotics in livestock and promoting the use of alternatives to enhance animal health and productivity without contributing to resistance [135].

### 4.5. Importance of AMR Policies and Regulations

#### 4.5.1. Protecting Public Health

By curbing the spread of resistant infections, these policies help maintain the efficacy of existing antibiotics, ensuring they remain effective for treating infections [114]. They reduce the burden of healthcare-associated infections, lowering morbidity and mortality rates [126].

#### 4.5.2. Promoting Sustainable Healthcare

Effective AMR policies contribute to the sustainability of healthcare systems by preventing the escalation of treatment costs associated with resistant infections [115,127]. They support the development of new antimicrobial agents and treatments, ensuring a continuous pipeline of effective therapeutic options [136].

#### 4.5.3. Enhancing Global Health Security

Coordinated international efforts to combat AMR enhance global health security by preventing the cross-border spread of resistant pathogens [114,118]. These efforts support the resilience of healthcare systems worldwide, improving their ability to respond to AMR-related challenges and emergencies [133].

#### 4.5.4. Encouraging Responsible Practices

Policies and regulations encourage responsible antibiotic use in both human and veterinary medicine, preserving the effectiveness of these vital drugs for future generations [137]. They promote best practices in infection control and prevention, reducing the incidence and spread of infections [129].

Global and national policies have been established globally to combat AMR through a diverse range of strategies, underscoring the critical importance of responsible antibiotic utilization, vigilant surveillance, comprehensive research, and effective infection control measures. Prominent international frameworks include the WHO Global Action Plan [114], which delineates a holistic approach to address AMR through enhanced awareness, robust surveillance systems, and the promotion of responsible antibiotic practices. Similarly, the European Union’s Action Plan on AMR [133] adopts a “One Health” paradigm to enhance surveillance and research initiatives throughout Europe.

At the national level, various countries have crafted specific action plans to mitigate AMR. For instance, the United States National Action Plan for Combating Antibiotic-Resistant Bacteria [127] prioritizes surveillance, stewardship, and research efforts within its healthcare system. Australia’s Antimicrobial Resistance Strategy (2015–2019) similarly emphasizes the necessity of infection control and stewardship [128].

Regional initiatives further bolster these efforts, as demonstrated by Canada’s Pan-Canadian Framework [138] and Japan’s National Action Plan [134], each tailored to address AMR through innovative surveillance and targeted measures. Additionally, collaborative endeavors such as the Global Antibiotic Research and Development Partnership [131] and the Global AMR Research and Development Hub [139] are dedicated to advancing the development of novel treatments for resistant infections on a global platform.

Countries as diverse as China [137], India [130], South Africa [135], and Thailand [140] have formulated national action plans that align with the WHO framework, implementing strategies meticulously tailored to their unique health infrastructures. The WHO AWaRe Classification [141] further supports the promotion of judicious antibiotic use worldwide by categorizing antibiotics in a manner that optimizes stewardship practices. Collectively, these policies signify a concerted international endeavor to alleviate AMR through integrated strategies that span the human, animal, and environmental health sectors.

## 5. Combatting AMR Through Innovations in Antibiotic Development and Strategic Enhancements

### 5.1. Mechanisms of Resistance

A fundamental aspect of developing new antibiotics is understanding the mechanisms by which bacteria resist current treatments. Bacterial resistance arises through several sophisticated mechanisms: enzymes that break down drugs, mutations that alter drug targets, efflux pumps that expel drugs from cells, and the formation of biofilms that shield bacteria from antibiotics [142,143]. To design effective new antibiotics, it is crucial to identify bacterial targets that are essential for the bacteria’s survival and less prone to resistance. This helps in targeting vital components of bacterial physiology while avoiding mechanisms that are likely to evolve resistance. Advances in genomics and proteomics have made it possible to uncover these targets with greater precision, laying the groundwork for novel antibiotic development [144]. The phenomenon of bacterial resistance to antibiotics is a multifaceted issue, characterized by several strategies that bacteria employ to evade the effects of these drugs. Understanding these mechanisms is vital when developing new antibiotics or improving existing treatments.

One of the primary ways bacteria resist antibiotics is through modifying the structure of antibiotic molecules. Bacteria can produce specific enzymes that chemically alter an antibiotic, thereby diminishing its ability to function effectively. For instance, aminoglycosides, which target bacterial ribosomes, often fall victim to such modifications. Enzymes like acetyltransferases, adenyltransferases, and phosphotransferases attach chemical groups to the antibiotic, changing its structure and preventing it from binding to its target, thus reducing its antibacterial potency [145]. Another significant resistance mechanism is limiting the penetration of antibiotics into bacterial cells. This is particularly prevalent in Gram-negative bacteria, which can alter their outer membrane to decrease their permeability [146]. By reducing the expression of porins—channels that allow antibiotics to enter the cell—bacteria can effectively prevent drugs like β-lactams and fluoroquinolones from reaching their targets within the cell, thereby reducing their efficacy [147].

Bacteria also utilize efflux pumps to resist antibiotics. These pumps are proteins embedded in the bacterial cell membrane that actively expel antibiotics from the cell, keeping the intracellular concentration of the drug low enough to avoid lethal effects [148]. Efflux pumps can be either highly specific to certain antibiotics or broad-spectrum, affecting multiple drug classes. This mechanism is a common cause of resistance to drugs like tetracyclines, macrolides, and fluoroquinolones, and the genes responsible for these pumps are often found on mobile genetic elements or integrated into the bacterial genome [149]. In addition to these strategies, bacteria can interfere with antibiotic action by altering or protecting their target sites. For example, mutations in the bacterial ribosomal RNA or associated proteins can prevent antibiotics from binding effectively, thereby neutralizing their intended effects [150]. Some bacteria produce proteins that shield these target sites from antibiotics. This protection mechanism is observed with tetracycline resistance proteins like Tet(M) and Tet(O), which dislodge the antibiotic from its binding site on the ribosome [151]. Similar protective mechanisms have been noted for resistance against fluoroquinolones and fusidic acid [151].

Mutations in the target sites of antibiotics are another common resistance strategy. These genetic changes can alter the structure of the target site, making it less susceptible to antibiotic binding. A well-known example is resistance to rifampin, a critical drug for tuberculosis treatment, which arises from mutations in the gene encoding RNA polymerase [152]. These mutations change the drug’s binding site, reducing its ability to inhibit the bacterial enzyme, and further contributing to bacterial resistance. Some bacteria acquire enzymes that chemically modify their target sites, reducing the binding affinity of antibiotics. For example, the methylation of ribosomal RNA by enzymes encoded by the erm genes results in resistance to macrolides [153]. Additionally, the chloramphenicol–florfenicol resistance (*cfr*) gene confers resistance to multiple antibiotic classes by methylating the antibiotic binding site on the bacterial ribosome [154].

Finally, bacteria can resist antibiotics by replacing or bypassing the target sites. In some cases, bacteria evolve alternative target sites that perform the same function but are not affected by the antibiotic. MRSA is a classic example, as it has acquired an alternative penicillin-binding protein that is not inhibited by methicillin [155]. Bacteria can also bypass the metabolic pathways that antibiotics target, as seen in resistance to trimethoprim-sulfamethoxazole, where overproduction of the enzyme dihydropteroic acid synthase allows the bacteria to survive despite the drug’s presence [156].

### 5.2. Design and Synthesis of New Antibiotics Against Resistant Microorganisms

In the face of the growing crisis of antibiotic resistance, the design and synthesis of new antibiotics have become a critical imperative for modern medicine [157]. As bacteria evolve and develop resistance to existing drugs, the need for novel antibiotics capable of overcoming these resistance mechanisms has never been more urgent [158]. The increasing challenge of antibiotic resistance, worsened by misuse, has driven the need for new antimicrobial agents. The World Health Organization highlights the urgent need for new antibiotics to target high-risk bacteria with limited treatment options [159]. Despite the critical need for new antibiotics, the past few decades have seen a stark decline in novel drug discoveries. This stagnation is partly due to pharmaceutical companies withdrawing from antibiotic research, driven by low financial returns, high costs, and the rapid development of resistance. High-throughput screening (HTS), a prevalent method for drug discovery, has proven ineffective in finding new antibiotics, with many large-scale efforts failing to yield viable candidates [160].

The creation of new antibiotics is essential in addressing the challenge of drug-resistant bacteria. Notable examples include Teixobactin, identified in 2015, which combats Gram-positive bacteria by disrupting their cell walls, reducing the likelihood of resistance. Lefamulin, approved in 2019, is used to treat drug-resistant Streptococcus pneumoniae by blocking protein synthesis. Zoliflodacin, currently being researched for resistant gonorrhea, interferes with DNA replication. Cefiderocol, a siderophore cephalosporin, is particularly effective against drug-resistant Gram-negative bacteria, including those resistant to carbapenems, by disrupting cell walls and enhancing iron uptake. These innovative drugs provide alternative treatments for persistent infections [161]. Although developing new antimicrobial agents is crucial in addressing AMR, the scientific community seems to lag in the rapid evolution of MDR bacteria. While discovering entirely new drugs is a logical strategy, the time and resources required are substantial [162].

One promising area is the rational design of antibiotics, which involves the use of computational models. Computational methods are increasingly essential for developing new antibacterial agents by modelling how drug modifications can enhance their effectiveness or combat resistance. In-silico modelling uses computer simulations to explore drug interactions and predict new candidates, such as antimicrobial peptides (AMPs) and natural compounds with potential antibacterial properties [163]. Fragment-Based Drug Design (FBDD) starts with small molecular fragments to create potent inhibitors for various targets [164]. For instance, β-lactam antibiotics are often rendered ineffective by enzymes like New Delhi Metallo-β-lactamase-1 (NDM-1), making it essential to develop new inhibitors. FBDD was used to identify potential NDM-1 inhibitors through the virtual screening of over 700,000 compounds, fragmenting the hits, and then testing them with NMR. The method identified 37 promising fragments, leading to the synthesis of Indenone 89, which effectively inhibited NDM-1 with a Ki value of 4 μM [165].

Furthermore, the exploration of novel antibiotic classes, such as AMPs, has gained attention. AMPs are small, diverse proteins either naturally found in various organisms or synthetically produced, known for their ability to fight bacteria, viruses, and fungi and modulate the immune system. They hold significant potential in tackling MDR bacteria due to their strong, fast-acting, and low-resistance nature [166]. Key examples include the human cathelicidin peptide LL-37 and colistin-based derivatives, both demonstrating effective antimicrobial properties [167]. However, AMPs face challenges such as their susceptibility to degradation in the body, thereby affecting their stability. Innovations like selectively targeted AMPs (STAMPs) are being developed to improve their targeting and effectiveness; however, further research is necessary to fully utilize them [168].

Another cutting-edge approach is the synthesis of hybrid antibiotics that combine the pharmacophores of two different antimicrobial agents. Recent efforts in antibiotic development emphasize hybrid antibiotics, Antimicrobial Peptide-Based Combinatorial Treatments (AMP-based combinations), and Metal Nanoparticle-Based Combinatorial Treatments (MNP-based therapies). Hybrid antibiotics aim to combat resistance by targeting multiple bacterial pathways simultaneously. Combining AMPs with antibiotics enhances their effectiveness and allows for lower dosages. Likewise, MNPs, particularly those based on silver, have been found to significantly boost the efficacy of existing antibiotics, especially against resistant bacteria. These combined approaches offer promising strategies for creating new antibacterial treatments to address the growing issue of antibiotic resistance [169]. For example, fluoroquinolones, a type of quinolone derivative, have become the most frequently prescribed antibiotics for treating a wide range of bacterial infections. Combining the quinolone structure with other antibacterial elements allows these hybrid compounds to target multiple drug mechanisms, helping to counteract resistance. As a result, quinolone hybrids serve as valuable models in the fight against drug-resistant pathogens [170].

### 5.3. Strategies to Enhance Efficacy and Reduce Resistance

Enhancing the efficacy of existing antibiotics and reducing the potential for resistance development are essential components of the fight against AMR. Several strategies have been proposed and are being actively investigated. These approaches include modifying the chemical structure of current antibiotics, employing combination therapies, utilizing drug-adjuvant pairings, developing new antibiotic derivatives, and exploring alternative treatments beyond traditional antibiotics.

One effective method to combat antibiotic resistance is by altering the chemical structure of existing antibiotics to enhance their potency against resistant bacteria. A prime example of this is the modification of vancomycin, which has been a reliable treatment against Gram-positive bacteria for nearly 60 years [171]. However, the emergence of resistance, such as in Vancomycin-Resistant Enterococcus (VRE), prompted researchers to modify vancomycin’s structure. These modifications have not only increased its effectiveness by 6000 times against resistant strains but also introduced new mechanisms of bacterial destruction, illustrating the power of chemical alterations in overcoming resistance [172].

Another essential tactic is the use of combination antibiotic therapies, which involves the simultaneous application of multiple antibiotics to reinstate bacterial susceptibility and enhance treatment success. This strategy can function through various mechanisms, such as targeting different bacterial pathways or attacking the same bacterial component through different mechanisms [173]. For instance, combination therapy has been successfully used in treating diseases like HIV/AIDS, malaria, and tuberculosis, significantly improving treatment outcomes. Additionally, sequential dose regimens, where antibiotics are alternated over time, can increase bacterial sensitivity. However, this approach requires careful monitoring to prevent adverse drug interactions that might reduce efficacy or cause toxicity [174].

The combination of drugs with adjuvants—molecules that enhance antibiotic activity—is another promising approach. Adjuvants themselves do not kill bacteria but can significantly boost the effectiveness of antibiotics when used together [175]. A well-known example is Augmentin^®^, a combination of amoxicillin and clavulanic acid, where clavulanic acid inhibits enzymes that would otherwise render amoxicillin ineffective. This combination has successfully delayed the development of resistance, and ongoing research aims to discover new adjuvants, such as avibactam, which has shown effectiveness against resistant bacteria like Pseudomonas aeruginosa [176].

In addition to these strategies, the development of new antibiotic derivatives, particularly aminoglycosides, plays a crucial role in addressing resistance. Aminoglycosides, including streptomycin, have been effective against a wide range of bacteria, but resistance due to aminoglycoside-modifying enzymes (AMEs) has become a significant problem [177]. To combat this, new aminoglycosides like plazomicin have been developed. Plazomicin has shown superior activity against MDR Gram-negative bacteria and MRSA, highlighting the importance of creating new antibiotics to tackle resistance [178]. 

## 6. Environmental and Ecological Perspectives

### 6.1. Environmental Reservoirs of Resistant Genes

The acquisition and transmission of AMR is primarily facilitated by certain reservoirs and hotspots of ARGs and ARB. As a result, these favorable environments hasten the process of AMR development and effectively support its dissemination. Recent reports have implicated wastewater and sludge from clinical settings, such as hospital sewage discharges, wastewater treatment plants (WWTP), pharmaceutical industries, and animal production sites in increasing the likelihood of human exposure through direct inhalation, ingestion, or consumption of contaminated food, or water, posing significant public health risks [179,180].

Clinical settings have a relatively high level of antibiotic consumption which has been impacted by the COVID-19 pandemic and are considered AMR hotspots. Ruzsa et al. [181] reported a 397% increase in the consumption of azithromycin between 2019 and 2020 in Hungary, with an expected high level of antibiotic effluents. Furthermore, hospital effluents are rich in patient-derived microorganisms that readily acquire ARGs and pose further danger [182]. Chng et al. [183] studied the colonization patterns of resistomes and the genomic characterization of AMR cassettes in a tertiary-care hospital. Their findings indicated persistence of MDR bacteria for extended periods which aim to opportunistically infect patients. A significant portion of the antibiotics within therapeutic doses are released into the environment through the improper disposal of medical and pharmaceutical important waste products, which facilitates the entry of antibiotic residues into the soil and groundwater. Siri et al. [184] investigated the prevalence of MDR bacteria in hospital wastewater and its receiving waters. The results from their analysis of untreated wastewater, aeration tanks, sedimentation tanks, effluent after disinfection, upstream canals, and downstream canals indicated a relatively high level of resistance after treatment, with an overall MDR rate of 33.3%. Hospital wastewater is an important wastewater hotspot for ARGs, and recent research has demonstrated that the patterns of ARGs in wastewater resemble those found in clinical settings [185]. Wastewaters are usually subjected to treatment plants in most areas; however, this has proven insufficient in the complete elimination of ARB and ARGs and have been implicated in the dissemination of antibiotic parent compounds and metabolites into receiving waters [186,187].

High concentrations of ARGs have been reported in livestock and poultry farm sites, which further intensifies the levels ARGs in surface water and soil [188]. A recent meta-analysis study of 460 studies showed that animal production sites are reservoirs of ARGs with therapeutic potential [189]. Additionally, slaughterhouses have been reported as a major hotspot for the development, progression, and transmission of AMR pathogens [190]. The quantification of ARGs and mobile genetic elements (MGEs) in slaughterhouse wastes in Al-Dewanyiah province, Iraq indicated that the waste from livestock serves as a potential hotspot for AMR in the environment [191]. Furthermore, manure from antibiotic-consuming animals, irrigation water, and soil in animal production sites are considered hotspots due to the high consumption of antibiotics in these areas. A study on the antibiotic resistance profile of *E. coli* isolates from lettuce, poultry manure, irrigation water, and soil in Ghana indicated high levels of resistance among isolates. Subsequently, microorganisms associated with farm animals develop AMR through constant exposure to antimicrobial residues and further spread of resistance through the transfer of ARGs.

Recent studies have reported high levels of ARGs in the soil. Gekenidis et al. [192] reported the contribution of tap water and surface water on overhead-irrigated chive plants from planting to harvest. Findings showed that water quality affects the quality of irrigated produce, as high levels of ARGs were detected in both water sources. Similarly, Kampouris et al. [193] reported a correlation between the relative abundance of ARGs and treated wastewater irrigation intensity which decreased during irrigation breaks and showed a high spread of ARGs during treated wastewater irrigation, relative to freshwater irrigation. These reservoirs and hotspots act not only as incubators for the emergence of AMR but also as transmission routes for the transfer of ARB and ARGs. Certain ecological factors influence the persistence and spread of AMR in the environment, which affects the amount, composition, and durability of ARB and ARGs in the environment.

#### 6.1.1. Climate

High temperatures have been linked with increased levels of AMR persistence. A recent study on AMR in river biofilms explored the effect of temperature on naturally occurring resistomes in the river. The results showed that higher temperatures increased the abundance of naturally occurring ARGs [194]. Again, Van Eldijk et al. [195] reported that environmental factors such as a small change in temperature can affect the mutation rate towards antibiotic resistance. Extending the scale and depth of monitoring, Li et al. [196] estimated that every 1 °C increase in average ambient temperature was associated with a 2.71% increase in the prevalence of *E. coli* resistance to ceftriaxone and cefotaxime, but persuasively concluded that, in addition to temperature levels, socioeconomic factors play supportive roles in this positive correlation.

Precipitation relates to a high level of relative humidity, which is concomitant with increments in ARGs abundances. Rainfall promotes conditions conducive for the horizontal transfer of ARGs [197]. Molecular evidence from a study conducted by Zahra et al. [198] on the prediction and interpretation of the prevalence of ARGs in beaches affected by urban wastewater discharge showed that water temperature, precipitation, and tide are relevant predictors of the abundance of ARGs at beaches. Overall, reports on the frequencies of ARGs during winter and summer indicated a significant influence of seasonality on the abundance of ARGs [199].

These results indicate a significant positive correlation between climatic factors and the occurrence of ARGs and ARB, which aligns with reports on the effect of sunlight on the abundance of ARGs and ARB. Azuma and Hayashi [200] reported that the irradiation associated with exposure to sunlight was found to inactivate a large group of target ARB after 5 h of exposure. Upon exposure to sunlight, certain antibiotics may deplete, which includes improperly disposed antibiotics or constituents of wastewater that contain metabolites which may cause mutations and a rise of AMR in the surrounding microbiome [201]. However, environmental factors that result in the death and decomposition of bacteria allow the release and spread of ARGs. Additionally, ARGs in the atmosphere are affected by wind speed, wind direction, and the height of the mixing layer, which play a significant role in their persistence and transport [202].

#### 6.1.2. Habitats

ARB and ARGs move through habitats and must overcome resilient barriers to develop niches and thrive. The abundance of ARGs across different habitats is based on varying habitat determinants. Consequently, different habitats pose different strategic barrier methods. Mostly, autochthonous communities in certain habitats with higher diversity, richness, and evenness may develop diversity-based resilience against ARG migration, which could have a significant impact on the likelihood of ARGs being horizontally transferred to the endemic microbiota [203]. Han et al. [204] assessed the behavior of various ARGs in paddy soil during the rice growth period. Results showed that the abundance of ARGs in non-flooded soil exceeded that of flooded soil during rice growth (decreased by 33.4%). Furthermore, the study demonstrated an effective reduction in the proliferation and dissemination of most ARGs in paddy fields via the dry–wet alternation in soil, indicating that a short break of dryness may reduce the incidence of ARGs transmission. Xu et al. [34] analyzed 291,870 records on the abundance of 290 ARGs from five types of habitats between 2013 and 2020. A statistically significant difference was observed in the persistence of ARGs in different habitats, with the sediment habitat accruing 43.64% and 32.40% of multidrug and aminoglycoside resistance genes, respectively. Additionally, a study of the ARGs in the surface water, sediment, and biofilm in a wastewater-impacted river in southern Germany showed that biofilm samples presented a relatively higher abundance of ARGs [205]. In another study, a notable pattern of resistome coalescence was observed within similar habitat types among the five habitats evaluated for ARGs [206].

Soil structure, high altitude, and water stratification have also been reported to significantly influence the presence, transport, and persistence of ARGs within an environment. A recent metagenomic analysis showed that conservation tillage, which decreases soil disturbance and improves its physical and chemical properties relative to conventional tillage practices, showed a decreased abundance of ARGs [207]. Consequently, soil structure and porosity determine the movement of pollutants, which alters the persistence of ARGs in the soil. Relative to altitude, high-altitude lakes and low-altitude lakes in Mountain Sangguniang in the Eastern Tibetan Plateau were evaluated for the abundance of ARGs; an abundance of ARGs was observed with high-altitude lakes, indicating atmosphere-mediated organic pollutant enrichment driven by the Indian monsoon due to elevation [208].

#### 6.1.3. Environmental Contaminants

Environmental pollutants have a discernible role in the rapid development of resistance in pathogens. Heavy metals may occur as a result of anthropogenic wastes and are inherent constituents of the earth’s crust that have significantly been linked with the persistence of ARGs and ARB. Komijani et al. [209] reported ARG abundance in lakes and wetlands in Iran and a strong correlation between the concentration of heavy metals, such as vanadium, with that of antibiotics. Similarly, Edet et al. [210] reported a high level of co-existence between MDR bacteria and heavy metals present in dumpsites. The coexistence between antibiotics and heavy metals creates a synergistic relationship that amplifies the abundance of ARGs in the environment by protein expression [49]. Pesticides and herbicide deposits in soils have also been implicated in the increasing levels of ARGs in soil. Liao et al. [211] showed that glyphosate, glufosinate, and dicamba used as herbicides increased cell membrane permeability and frequency in conjugation of MGEs, which facilitates an ease in the uptake of ARGs among bacteria. On the same scale, co-contamination of topramezone and polymyxin reportedly resulted in elevated levels of MGEs and ARGs [212], and fungicides have been shown to alter the microflora within the gut of soil animals and enrich ARGs [213]. Additionally, molecular evidence shows that soils contaminated with carbon-rich compounds and microplastics affect the persistence of ARGs and ARB. Maurya et al. [214] reported the abundance of ARGs related to tetracycline, sulfonamides, aminoglycosides, ampicillin, and fluoroquinolone resistance in polyaromatic hydrocarbon (PAH)-contaminated environments. Biochar serves as an enabler of ARG transfer [215]. Microplastics serve as a colonization surface for most resistomes in river microbiomes. These microenvironments seemingly absorb toxic pollutants and further assist the transport of ARGs [216,217] and promotes environmental selection pressure [218].

### 6.2. Impact of Human Activities

Anthropogenic activities act as a significant driver in the increasing burden of AMR. These activities include the excessive and indiscriminate consumption of antibiotics, inappropriate disposal of antimicrobials, prophylactic use of antibiotics in food production, pollution, and poor waste management, which are disproportionately higher in low- and middle-income countries and allow the seepage of ARGs into the soil and surface and ground waters, facilitating their transmission through the food-supply chain and posing serious public health risks [219].

The prophylactic use of antibiotics in agriculture has raised serious concerns in recent times due to the increased purchase of antibiotics from local stores to feed animals without clinical signs of diseases. In the 1950s, the rising demand for food put pressure on the food industry, resulting in the use of antibiotics in food animals to boost production for economic gains. Although this practice was banned in some countries in the 2000s, farmers continue to use antibiotics indiscriminately for prophylactic purposes and as growth promoters to meet the high demand for agricultural products and profit goals. This ongoing use has resulted in the presence of resistomes in river reservoirs of resistance. In various regions of Africa, the use of antibiotics in livestock has been frequently reported. A recent study in Kenya reported that 92.7% of 175 cattle farms primarily used antibiotics for prophylactic purposes [220]. Similar trends have been reported across most low- and middle-income countries [221,222,223]. Evidence from current research suggests that more antibiotics are administered by farmers, majorly as prophylaxis, than for any other reason [223,224,225]. The continuous use of antibiotics in sub-therapeutic doses as growth promoters, feed enhancers, and prophylaxis has been reported as a significant contributor to the increasing levels of AMR [226]. Again, to increase the shelf life of animal products, antibiotics are being added directly to raw milk by vendors In Ethiopia [227]. Studies have further traced the ‘silent pandemic’ to the limited awareness and degree of misunderstanding about the problem of indiscriminate antibiotic use, AMR, and its consequences on health and economy among farmers [228,229]. ARGs and ARB extend into the broader environment through fertilizers made from manure, contaminating waterways and food produce that facilitate direct contact with humans [230]. Trends observed by Smith et al. [231] in an assessment of livestock farms in England show trends of AMR across the farms did not have corresponding trends in their antibiotic use, thereby suggesting the role of external factors in the persistence of AMR, which may involve the presence of environmental ARGs present in farm sites majorly because of direct human activities.

The combination of poverty, poor regulations, and lack of knowledge has significantly led to a high rate of dissemination and development of AMR. The misuse and overuse of antimicrobials, for example, by stockpiling leftovers for future use, transferring prescriptions, and the circulation of counterfeit drugs by individuals and manufacturing companies with the wrong ingredients and incorrect dosages, are worsened by poor hygiene and pollution. This allows AMR to thrive, especially in low- and middle-income countries. The improper disposal of waste containing therapeutic and sub-therapeutic doses of non-metabolized antibiotics and their residues is a major driver in the development and transmission of AMR in the environment. Antibiotics are indiscriminately consumed in high quantities, with most of them undergoing only partial metabolism within the human body. The unmetabolized parts are then excreted into the environment, thereby contributing significantly to antibiotic pollution. Poor sanitation and hygiene have been reported in different studies as a key driver of AMR caused by human activities. For example, in areas with poor hygiene practices, synanthropic filth flies serve as a vehicle for the localized transport of ARB and associated ARGs from human feces [232]. In certain areas, humans use a single water source for defecating, fishing, bathing, and carrying out domestic chores. In 2022, the WHO estimated that at least 1.7 billion people used a drinking water source contaminated with feces, which may cause illness and indirectly affect antimicrobial consumption, as well as transmit ARB and ARGs [233]. The improper release of leftover spent containers and packages or expired products into the environment contributes to the generation of waste that contains ARB or can lead to the development of AMR [234]. Likewise, significant quantities of solid waste such as over-the-counter antibiotics, prescription-only-antibiotics, contaminated gloves, masks, syringes, dusters, rags, mops, needles, etc., and liquid wastes, including wastewater and chemicals generated from hospitals, households, and pharmaceutical manufacturing industries, cause selective pressure on the bacteria present in that environment, which results in the development of various resistance mechanisms that act as defense strategies against antibiotics, enabling them to survive in antibiotic-loaded environments and trigger the development of AMR, which poses risks to food safety and public health [235].

## 7. Integrated Strategies to Combat AMR

### 7.1. Multidisciplinary Approaches

Containing and controlling AMR requires coordinated collaboration across various disciplines, including scientists (such as microbiologists, chemists, biochemists, and biologists) and public health experts, and sectors such as healthcare, agriculture, finance, trade, education, and NGOs at both national and international levels. Combating AMR requires a multidisciplinary collaborative approach involving contributions from the biological and social sciences to address it. To fully understand the AMR problem, one must consider social disciplines like economics and politics. Although the link between these fields and AMR might seem unlikely, decisions such as the choice of antibiotics are often based on cost-effectiveness, which is an economic consideration [236]. National public health bodies typically set treatment guidelines, resulting in a uniform national strategy where the same antibiotic is used across an entire region, thereby providing the ideal environment for the emergence of AMR when treatment regimens are not followed as it has been highlighted that using a single medication within a population encourages the development of AMR when the medication is abused [237]. To tackle this issue, public health experts and scientists can collaboratively implement informed prescription practices by tailoring antibiotics to individual patients, thereby reducing the selection pressure on a particular antibiotic and minimizing resistance development. Primarily, doctors and veterinarians often employ broad-spectrum antimicrobials due to functional limitations in quickly and accurately diagnosing infectious diseases, their causative organisms, and the susceptibility profile of pathogens [238]. Scientists and public health experts can collaborate to tackle this by developing targeted therapies, designing combination treatments using narrow-spectrum antibiotics, regulating the availability of antimicrobials as over-the-counter drugs (especially in developing countries), and establishing guidelines to ensure precise antibiotic use [239]. This can be achieved by conducting joint research, sharing data, and creating educational campaigns to promote responsible antibiotic prescription and usage, ultimately reducing AMR.

### 7.2. Development of Comprehensive AMR Mitigation Strategies

The continuous increase in the global human population has led to an increased demand for high-quality animal products, leading to the use of animal growth promoters in farm animals to sustain production and keep up with demand [240]. However, the widespread assumption is that the use of antibiotics in food animals for growth promotion and disease treatment and prevention is linked to the global spread of AMR [241]. When animals are continuously exposed to these growth promoters, bacteria in their bodies can evolve resistant genes to the antimicrobial agents in these growth promoters through mechanisms such as horizontal gene transfer or selective pressure [242]. There exists a potential for these resistant bacteria or their genes to be transferred to humans through the consumption of these animal products or water contaminated by animal wastes from the farm environment [59], leading to the development of AMR in humans and the potential compromise of the efficacy of antimicrobial therapy in both veterinary and human medicine [240]. As a result, it is critical to control the use of antibiotics for the stimulation of growth in animals, manage the use of antibiotics that are clinically significant, and optimize the dosage and duration of current antibiotic therapies [59]. Veterinary prescription guidelines should be mandatory, particularly for antibiotics used to treat infectious diseases in both humans and animals. Developing protocols to verify the prudent administration of antibiotics, setting goals for monitoring advancements, and formulating approaches for employing veterinary antibiotics are very crucial [66]. In the United States of America, for instance, the use of antibiotics as growth promoters in agriculture has been regulated by the FDA by classifying the two categories of antibiotic usage in their veterinary feed directive (VFD) regulations as therapeutic and non-therapeutic [243]. These regulations change how certain over-the-counter antimicrobial drugs are classified and ban their use in animal production, while also restricting the use of VFD drugs in animal feed to those prescribed by a licensed veterinarian based on a VFD order [244]. This regulation implies that some over-the-counter antimicrobial drugs such as tetracyclines like oxytetracycline and chlortetracycline were reclassified as VFD drugs, requiring veterinary supervision for their use according to a VFD order. The FDA also mandated that antibiotics in animal feed be clearly labelled for their specific approved uses, such as growth promotion or disease treatment, and ensured compliance through inspections and enforcement [244]. These regulations aim to balance the need for effective disease management and growth promotion in livestock to reduce the risk of antibiotic resistance affecting both animals and humans.

As demonstrated by Mir et al. [245], antibiotic usage in food animals may not be the only primary source of antibiotic resistance in grazing beef cattle operations. The study found that cefotaxime-resistant bacteria were present in 4.5% to 30% of cattle across seven herds that had never been treated with cefotaxime, suggesting that AMR microorganisms can colonize beef cattle without antibiotic exposure, likely originating from environmental sources. To mitigate the rise of AMR in grazing beef cattle operations, strategies should include enhancing biosecurity measures to prevent environmental contamination, monitoring water and soil quality for resistant bacteria [241], promoting proper manure management, and implementing rotational grazing to reduce pathogen build-up [241,246]. These strategies underline the significance of a One Health approach—a collaborative, multi-sectoral strategy for addressing AMR that emphasizes the interconnection of human, animal, and environmental health. This strategy intends to strengthen surveillance, antimicrobial stewardship, and education and awareness across every sector to successfully combat the spread of AMR. The One Health concept aims to provide a holistic response to AMR concerns by combining efforts from human healthcare, animal medicine, and environmental science, eventually protecting public health, ensuring food safety, and conserving effective infection treatment options.

Antimicrobial stewardship (AMS) is a coordinated strategy aimed at optimizing the use of antimicrobial medications to enhance patient outcomes, reduce resistance, lower antibiotic costs, and minimize adverse effects. This approach is essential in the fight against AMR [247]. This can be achieved through several key strategies. First, evidence-based guidelines for the appropriate use of antimicrobials should be developed and implemented [238]. Second, protocols for diagnosing infections and selecting the appropriate antimicrobial therapy need to be established [247]. Additionally, ongoing education and training for healthcare providers regarding proper antimicrobial use and resistance prevention are essential. It is also important to educate patients about the significance of completing prescribed courses and the risks associated with misuse [238]. Moreover, ensuring that antimicrobials are prescribed at the correct dose and duration will help maximize efficacy and minimize resistance. Finally, when appropriate, favoring narrow-spectrum antimicrobials over broad-spectrum ones can effectively target specific pathogens while reducing collateral damage to the microbiome [248]. These could encompass public health campaigns, hospital-based programs, National Action Plans (NAPs), and the Global Action Plan (GAP), including awareness initiatives like World Antimicrobial Awareness Week (WAAW) [249,250]. The GAP, for instance, highlights the importance of improving infection prevention and control measures through effective sanitation, vaccination programs, and stringent hygiene practices in healthcare settings, which aids in preventing the spread of infections and reducing the need for antibiotics [238]. For example, the success of hand hygiene campaigns in hospitals has been linked to reduced rates of healthcare-associated infections and, consequently, lower demand for antibiotics [251].

Hospital-based AMS programs can aid in managing the prudent use of antimicrobials to combat AMR, tailoring approaches to local resources and contexts, focusing on prescribing guidelines, monitoring use, and educating healthcare workers [252]. Outpatient AMS programs similarly promote appropriate antibiotic use in clinics and doctors’ offices through the education of patients and healthcare workers, and monitoring of prescription patterns [247]. For most bacterial illnesses, the typical course of antibiotic therapy is usually too lengthy and is not supported by evidence. Reducing the duration of typical antimicrobial regimens is therefore a straightforward and secure way to lower the use of antibiotics [253]. Recent infectious diseases pharmacotherapy has focused on defining the shortest effective therapy duration for various infections. In a study by Teshome et al. [254], over 7000 ICU patients were evaluated, and it was found that each additional day of antipseudomonal beta-lactam therapy increased the risk of new beta-lactam resistance, highlighting the importance of minimizing therapy duration to reduce AMR.

Surveillance, which is the systematic monitoring and analysis of the occurrence and spread of AMR pathogens, is a key priority of the GAP on AMR, aiding countries in collecting data on AMR and antimicrobial use to enhance patient outcomes, inform policy, and recommend interventions [114]. AMR surveillance typically involves collecting data on infections and antimicrobial use from clinical settings and laboratories, performing resistance testing on bacterial isolates, and analyzing trends and patterns in resistance [255]. These data are reported in national and regional summaries and used to provide feedback to healthcare practitioners. Surveillance systems, including local, national, and international, help coordinate data collection and inform public health interventions and infection control measures to address AMR [238]. Comprehensive surveillance of antimicrobial use is essential to prevent antibiotic-resistant bacteria and monitor AMR, helping to assess the problem’s scale, track new and spreading resistances, and determine trends and outbreak causes [247]. Surveillance reports have shown that between 2000 and 2015, global antimicrobial use expressed in defined daily doses (DDD) increased by 65% (21.1–34.8 billion DDDs), and if unregulated, this could rise to 42 billion DDD by 2030 [256]. Effective surveillance systems are crucial for monitoring AMR across animals, humans, agriculture, and the environment, helping to identify the presence of AMR in specific populations, including food-producing animals like chickens, pigs, cattle, and goats, as well as their products [257,258]. The large-scale use or misuse of antibiotics in food-producing animals and humans may promote the development of AMR, necessitating surveillance programs [114,257].

Various antimicrobial surveillance programs have been instituted over the years. An example is the GLASS, which was created in 2015 to encourage the monitoring of AMR in bacteria that cause common infections in humans and food-producing animals, as well as the development of strategies such as collecting and analyzing data from various sources, including epidemiological, population-level, clinical settings, and laboratories, to monitor resistance patterns and guide interventions [114]. Additionally, the FAO prioritizes monitoring AMR in healthy food-producing animals to ensure food safety, recommending the surveillance of indicator microorganisms like *E. coli* and *Enterococcus* spp., helping to identify resistance patterns in bacteria [115]. These strategies help detect emerging resistance trends and inform measures to mitigate the impact of AMR on both human health and food safety. In addition, efficient laboratory practices are essential for effective AMR surveillance, as they involve detecting, isolating, and monitoring resistant pathogens. Laboratories also perform antimicrobial susceptibility testing and use advanced molecular techniques, such as PCR and whole-genome sequencing, to understand the genetic basis of AMR [259]. These practices are integral to strategies for mitigating AMR, as they provide critical data needed to identify resistance patterns, inform treatment decisions, and develop targeted interventions to control the spread of resistant infections.

### 7.3. Policy and Regulatory Frameworks

To guarantee that everyone has access to the continuous, successful treatment and prevention of infectious diseases with quality-assured, safe, and effective medicines—while using antimicrobials responsibly—the World Health Assembly’s “Global Action Plan (GAP)” outlined five strategic objectives: to improve the awareness and understanding of AMR; to strengthen knowledge through surveillance and research; to reduce the incidence of infection; to optimize the use of antimicrobial agents; and to ensure sustainable investment in countering AMR strategies to be implemented within the next five to ten years [114]. In 2019, the WHO, the International Federation of Pharmaceutical Manufacturers and Associations, the European Investment Bank, and the Wellcome Trust launched the AMR Action Fund, a nearly USD 1 billion initiative aimed at advancing promising antibiotics through the later stages of clinical trials and developing 2–4 new antibiotics by 2030. This fund aims to address unmet medical needs, advance medical science, slow resistance emergence, and support appropriate patient access by creating favorable market conditions for sustainable investment in the antibiotic pipeline [260]. So far, the fund has supported biotech companies in navigating the complex and costly process of bringing new antibiotics to market [261]. However, while this fund is a significant step forward, it is not a complete solution; substantial government support is still needed to implement long-term market reforms and incentives, as the current economic model for antibiotic development is inadequate, discouraging investment due to low potential returns.

Many countries have developed National Action Plans (NAPs) on AMR, aligned with the GAP objectives, to monitor and address antimicrobial resistance using a “One Health” approach [249]. For instance, The United Kingdom’s current NAP, which is based on its 2013–2018 strategy, was created in consultation with a wide range of stakeholders from a variety of sectors, and is linked to international frameworks and plans for action aimed at addressing AMR both domestically and internationally, with the ultimate goal of ensuring the realization of the 20-year vision on AMR; enabling the effective containment and management of resistance escalation [261]. To achieve this goal, the UK government has taken steps such as implementing antimicrobial stewardship initiatives across the National Health Service to ensure that antibiotics are prescribed appropriately and only when necessary, including regular training for healthcare professionals and developing guidelines for the prescription of antibiotics [262]. The UK government also established surveillance systems such as the English Surveillance Program for Antimicrobial Utilisation and Resistance (ESPAUR), which monitors antibiotic use and resistance patterns [263]. In 2014, the Canadian government introduced a federal framework involving a One Health approach to combat AMR through coordinated efforts from agencies like the Health Canada (HC), Public Health Agency of Canada (PHAC), Agriculture and Agri-Food Canada (AAFC), the Canadian Food Inspection Agency (CFIA), the Canadian Institutes of Health Research (CIHR), Industry Canada (IC), and the National Research Council (NRC) to prevent, limit, and control the emergence and spread of AMR in humans, animals, and the environment through optimized treatment practices, enhanced surveillance of antimicrobial use and resistance, and prevention of AMR transmission [264]. Through agencies such as the Canadian Antimicrobial Resistance Surveillance System (CARSS) and Canadian Integrated Program for Antimicrobial Resistance Surveillance (CIPARS), the Canadian government has been able to collect and analyze AMR and antimicrobial use data from various sources, including humans, animals, and food; and specifically track AMR trends in bacteria across these sources [265]. Building on the 2015–2019 strategy, the Australian government has unveiled the National Antimicrobial Resistance Strategy: 2020 and beyond, a 20-year action plan that synchronizes with the GAP of the WHO to tackle AMR concerns both nationally and internationally through actions such as improving infection prevention and control practices in healthcare settings and enhancing antimicrobial stewardship in both humans and animals [266]. However, when compared with the intervention tactics of developed countries, sub-Saharan African countries are not as equipped for AMR because they lack real-time surveillance, monitoring, and regulatory standards [267]. A study by Iwu and Patrick [268] showed that GAP implementation in the WHO African region is generally inadequate, with Ethiopia, Kenya, and Zambia performing relatively better, but most countries scoring poorly, which may be due to economic, sociological, technological, industrial, and ecological challenges.

In 2017, the WHO created the Access, Watch, and Reserve (AWaRe) classification to guide more sustainable and rational antimicrobial use, aiming to reduce AMR’s development and spread [269]. The AWaRe classification is a framework designed to guide the appropriate use of antibiotics based on specific conditions and recommended drugs, informing policy and practice in AMS programs to optimize antimicrobial use [247]. This framework categorizes antimicrobials based on their range of activity and their likelihood to contribute to AMR [270]. Narrow-spectrum antimicrobials that are effective against a particular class of pathogens and less likely to cause resistance are found in the “Access” group. Considering how important these antimicrobials are for treating a variety of common diseases, they should also be readily available, reasonably priced, and of high quality [270]. Broader-spectrum antibiotics with a higher potential for resistance are included in the “Watch” group [270]. Because overuse and misuse of these antimicrobials offer serious hazards for the formation and spread of AMR, their usage should be continuously regulated. Finally, the “Reserve” group consists of last-resort antibiotics that, if all other treatments are ineffective, should be administered judiciously and sparingly for serious infections [270]. However, while the AWaRe classification system has made significant strides in promoting the rational use of antibiotics and mitigating AMR, challenges remain in its effective implementation. Considering that the framework helps in setting guidelines, its major success depends on consistent adherence to these guidelines across healthcare settings. Issues such as the availability of essential antibiotics, varying levels of regulation and enforcement, and the need for continuous education and monitoring pose ongoing challenges [247]. Improving access to appropriate antibiotics, ensuring their quality and affordability, and strengthening stewardship practices are crucial for enhancing the impact of the AWaRe classification and combating AMR more effectively.

## 8. Conclusions

Addressing AMR is a multifaceted and challenging issue, necessitating a concerted, multidisciplinary approach that involves collaboration from the scientific, public health, agricultural, and policy sectors. The growing concern of AMR, fueled by factors including the abuse of antibiotics in human medicine and animal husbandry, over-reliance on broad-spectrum antibiotics, and inadequate regulatory framework implementation, highlights the critical need for comprehensive preventive and therapeutic strategies. Integrated approaches, such as the development of targeted therapies, improved antimicrobial stewardship, and effective surveillance systems, are crucial in preventing the spread of AMR.

It is imperative that all stakeholders commit to immediate actions that prioritize antibiotic stewardship and responsible use across all sectors. The effective implementation of national and international action plans guided by a One Health approach is crucial for promoting responsible antibiotic use, enhancing infection prevention and control practices, and maintaining sustained investment in antibiotic research and development. Future research should focus on identifying novel antimicrobial agents, optimizing the use of existing antibiotics, and exploring alternative therapies, such as phage therapy and immunotherapies, to combat resistant strains. Furthermore, research should focus on the identification of sustainable agricultural practices aimed at minimizing antibiotic utilization. This includes the enhancement of animal husbandry techniques, the adoption of integrated pest management strategies, and the implementation of crop rotation systems designed to decrease reliance on chemical inputs. Additionally, investigating the efficacy of probiotics and alternative treatments to antibiotics in livestock management holds promise for significantly reducing the risks associated with AMR. Moreover, fostering collaboration among researchers, agricultural practitioners, and policymakers is essential in the formulation and dissemination of best practices within the realm of sustainable agriculture that prioritize both animal welfare and environmental health. Achieving success in the reduction or mitigation of AMR will necessitate a multifaceted approach that combines robust legislative frameworks, public awareness campaigns, and ongoing training for healthcare professionals and agriculturalists. The successful reduction or mitigation of AMR involves a combination of solid legislative frameworks, public awareness initiatives, and continuous training for healthcare professionals.

The Global Action Plan and efforts such as the AMR Action Fund represent important steps forward, but long-term solutions will require sustained governmental backing and international collaboration. Collective global action must include the establishment of robust monitoring systems for antibiotic use and resistance patterns, coupled with public health campaigns that inform communities about the consequences of antibiotic misuse. The challenges posed by AMR are enormous, but with concentrated efforts and a dedication to innovation and collaboration, it is possible to ensure antibiotic efficacy for future generations while avoiding the life-threatening consequences of untreatable infections.

## Figures and Tables

**Table 1:** Studies on human health risks associated with AMR infections.

Aspect of AMR	Summary of Studies	Key References
Antibiotic Use in Healthcare and Agriculture	Antibiotic consumption remains high globally, with variations across regions. In Europe, community consumption varies widely (11.3 to 31.9 DDD per 1000 inhabitants per day), while hospital use is substantial (2.0 DDD per 1000 inhabitants). In the US, outpatient use is prevalent (258 million courses annually).	[100]
Factors Contributing to Resistance	Overuse and inappropriate use of antibiotics in both community and hospital settings drive resistance. Misconceptions and self-medication practices also contribute significantly to the problem, particularly in developing countries.	[101]
Case Studies: *S. aureus*, NTS, *K*. *pneumoniae*	Specific pathogens like MRSA, NTS, and ESBL-producing *K. pneumoniae* show high resistance rates globally, affecting treatment efficacy and healthcare outcomes.	[102]
Global Burden and Mortality	Drug-resistant infections in 2019 contributed to an estimated 4.95 million deaths globally, with 1.27 million directly attributable to AMR. AMR ranks as a leading global health concern, third in causes of death following ischemic heart disease and stroke in a hypothetical scenario without infections.	[103]
Environmental Spread of Resistance	Environmental factors, such as wastewater contamination, contribute to the spread of antibiotic-resistant bacteria, highlighting the need for environmental stewardship and surveillance.	[104,105]
Public Health and Economic Impact	Antibiotic-resistant infections in the US result in over 2 million illnesses and 23,000 deaths annually, with associated costs exceeding USD 55 billion, including direct healthcare expenses and lost productivity. In Europe, resistance contributes to 25,000 deaths annually, with estimated costs exceeding 1.5 billion euros.	[106]
Tuberculosis and Drug Resistance	MDR-TB and XDR-TB pose significant challenges to global TB control efforts, with high mortality rates and limited treatment options. The development of resistance is linked to treatment non-compliance and inadequate drug regimens.	[107]
Regional Disparities in AMR Burden	Low- and middle-income countries (LMICs), particularly in sub-Saharan Africa and South Asia, face higher AMR-related mortality rates. Factors include critical infections, inadequate healthcare infrastructure, and inappropriate antibiotic use.	[108]
Study for Monitoring AMR Trends (SMART)	Surveillance data are available through reports in scientific and medical journals, as well as freely available on hosts’ websites (e.g., EARS-net).	[12]
Impact on Specific Populations and Settings	AMR significantly affects vulnerable populations (e.g., cancer patients, transplant recipients, neonates) and specialized settings (e.g., neonatal intensive care units, surgical wards), leading to increased morbidity and mortality.	[109]
Impact of Resistance to Specific Antibiotics	Resistance to fluoroquinolones and β-lactam antibiotics is prominent across various pathogens, contributing significantly to mortality rates and treatment challenges.	[110]
Challenges and Strategies	Intervention strategies include infection prevention, vaccination, reducing unnecessary antibiotic exposure, promoting appropriate antibiotic use, and developing new antibiotics. Crucial for regions with high AMR burdens and limited healthcare resources.	[111]
Pathogens Contributing to AMR	Six major pathogens (*E. coli*, *S. aureus*, *K. pneumoniae*, *S. pneumoniae*, *A. baumannii*, *P. aeruginosa*) are significant contributors to AMR burden. These pathogens are prioritized by WHO due to their resistance profiles and global impact.	[112]
UK Government Actions on AMR	The UK government has implemented a 20-year vision to control and contain AMR by 2040, supported by 5-year national action plans. Achievements include reduced antibiotic use in food-producing animals, improved surveillance systems, and new payment schemes for antibiotics on the NHS.	[113]
